# Metabolomics integrated elementary flux mode analysis in large metabolic networks

**DOI:** 10.1038/srep08930

**Published:** 2015-03-10

**Authors:** Matthias P. Gerstl, David E. Ruckerbauer, Diethard Mattanovich, Christian Jungreuthmayer, Jürgen Zanghellini

**Affiliations:** 1 Austrian Centre of Industrial Biotechnology, Vienna, Austria, EU; 2 Department of Biotechnology, University of Natural Resources and Life Sciences, Vienna, Austria, EU

## Abstract

Elementary flux modes (EFMs) are non-decomposable steady-state pathways in metabolic networks. They characterize phenotypes, quantify robustness or identify engineering targets. An EFM analysis (EFMA) is currently restricted to medium-scale models, as the number of EFMs explodes with the network's size. However, many topologically feasible EFMs are biologically irrelevant. We present thermodynamic EFMA (tEFMA), which calculates only the small(er) subset of thermodynamically feasible EFMs. We integrate network embedded thermodynamics into EFMA and show that we can use the metabolome to identify and remove thermodynamically infeasible EFMs during an EFMA without losing biologically relevant EFMs. Calculating only the thermodynamically feasible EFMs strongly reduces memory consumption and program runtime, allowing the analysis of larger networks. We apply tEFMA to study the central carbon metabolism of *E. coli* and find that up to 80% of its EFMs are thermodynamically infeasible. Moreover, we identify glutamate dehydrogenase as a bottleneck, when *E. coli* is grown on glucose and explain its inactivity as a consequence of network embedded thermodynamics. We implemented tEFMA as a Java package which is available for download at https://github.com/mpgerstl/tEFMA.

Constraint-based reconstruction and analysis methods have been proven to be valuable tools in gaining system wide understanding of cellular metabolism^1^,^2^,^3^. These methods use mathematical reconstructions of metabolism together with (physiochemical, thermodynamical, environmental, *etc.*) constraints to derive their predictions. Based on a steady-state analysis of a stoichiometric matrix (i.e. an ordered collection of the stoichiometric coefficients of all contributing biochemical reactions) these methods allow for phenotypic predictions from genotype data^4^. Here we focus on a method called elementary flux mode (EFM) analysis (EFMA).

EFMA decomposes the stoichiometric matrix into non-decomposable, non-zero steady-state pathways, called EFMs^5^. EFMs are an important structural concept as any metabolic steadystate can be expressed as a non-negative, linear superposition of EFMs. Thus, the complete set of EFMs fully characterizes the available metabolic space. This comes at the price of a dramatically increased computational effort which goes beyond current capabilities for large, genome scale metabolic models^6^. A pessimistic upper bound for the number of EFMs in a network was derived^7^, but the exact computational complexity is not yet known^8^.

Regardless of the theoretical challenges, several software tools are available and allow the calculation of the full set of EFMs at least in small or medium scale (metabolic) models^6^. Very recently, a massively parallelized approach to completely enumerate EFMs in large-scale networks was presented^9^. For large genome-scale networks particular EFMs, but not all can be calculated. Various strategies ranging from calculating the shortest EFMs^10^ to different sampling approaches^11^,^12^ have been proposed. Recently, Pey and Planes^13^ identified a small subset of biologically interesting EFMs in a genome-scale model. Similarly, Kelk *et al.*^14^ search for all EFMs, which span the optimal solution space as defined by a flux balance analysis. Despite all these advances a full enumeration of EFMs in large genome-scale models is as yet out of reach.

EFMA utilizes stoichiometric information only. Yet, many of the topologically feasible EFMs are infeasible *in vivo* as they are in opposition to other constraints that have not been accounted for, like known regulatory mechanisms^15^,^16^ or thermodynamic properties of biochemical reactions^17^. Incorporating thermodynamic constraints allows us to draw conclusions on the directionality and feasibility of reactions and whole pathways. A single biochemical reaction occurs spontaneously only if its change in Gibbs energy is negative. To derive thermodynamic constraints for the whole network, metabolite data are particularly useful as they determine the Gibbs energy surface.

Here we present a novel computational tool – thermodynamic EFMA (tEFMA) – which integrates the cellular metabolome into the EFMA. This allows us to verify the thermodynamic feasibility of EFMs already during the runtime of the EFMA and curbs the explosion of the number of EFMs without losing any biologically relevant EFMs. Computationally, our new approach successfully tackles the major bottleneck of double description based EFMA by strongly reducing computational costs, both in terms of runtime and resource consumption. Biologically, tEFMA allows the identification of infeasible pathways based on an unbiased analysis derived from first principles. More specifically, tEFMA correctly predicts the inactivity of the glutamate dehydrogenase (GDH) in *E. coli* under glucose saturated conditions.

## Methods

### Theory

The stoichiometry of a metabolic network with *m* (internal) metabolites and *r* reactions can be represented by an *m* × *r* matrix, ***S***. At steady-state all flux distributions, ***v***, obey ***Sv*** = 0 and ***v***_irrev_ ≥ 0, where ***v***_irrev_ is a sub-vector of ***v*** containing only irreversible reactions. We assume that the network contains only irreversible reactions, as any reversible reaction can be split into an irreversible forward reaction and an irreversible backward reaction. Of particular interest are so called EFMs, *e_i_*^5^. These are steady-state flux distributions of minimal support fulfilling all irreversibility constraints. Minimal support means that if any of the contributing, i.e. supporting reactions (*v_i_* > 0) is omitted, the remaining reactions can no longer carry a steady-state flux. Geometrically, the EFMs (in a network of irreversible reactions) can be regarded as extreme rays, i.e. edges, in a convex polyhedral cone^18^. Several EFM-enumeration strategies are known^6^. Here we utilized the binary null-space algorithm^19^, which we will briefly outline below.

The binary (null-space) approach represents EFMs as binary bit vectors of the supporting reactions. These bit patterns are generated iteratively. Starting from an initial solution matrix (typically the kernel of ***S***) each row of this matrix is processed and converted to binary form. For each row (i.e. reaction) intermediate EFMs (that are the columns of the matrix) are combined such that their fluxes are nonnegative and therefore convertible to a bit representation and added to the matrix. New intermediates are added to the quickly growing list of intermediate EFMs if they are not a superset of any other intermediate EFMs. The iteration stops if all reactions are processed and the intermediate EFMs are fully converted into binary format. The remaining intermediate EFMs are then, in fact the EFMs. A step by step example can be found in the supplementary material, section “Proof of safe removal of thermodynamically infeasible EFMs” on page S-14.

An important feature of the binary approach is the inheritance of flux activity. When a reaction is converted to binary form and found to be active in an intermediate EFM, all progenies of this EFM will have an active flux in that reaction too^19^. This property is key to our approach. Based on metabolomics data we identify thermodynamically infeasible flux patterns and drop the associated modes from the list of intermediate EFMs, as all their possible offspring will be supersets of these infeasible flux patterns, and therefore will remain infeasible too. Thus, removing thermodynamically infeasible modes has no impact on any feasible (intermediate) EFM. Here, network embedded thermodynamic (NET) analysis^20^ is used to identify thermodynamically infeasible EFMs. NET analysis is briefly reviewed below.

The second law of thermodynamics states that at constant pressure any biochemical reaction, *i*, proceeds spontaneously only in the direction of the negative Gibbs free energy of reaction Δ_r_*G_i_*. As our network contains only irreversible reactions this translates into
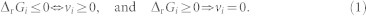
Δ_r_*G_i_* can be estimated from the Gibbs free energy of formation, Δ_f_*G_j_*, of the contributing reactants, *j*:
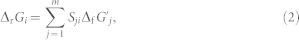

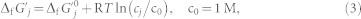
where *S_ji_* represents the stoichiometric coefficient of metabolite *j* in reaction *i* and 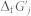 is used to denote the transformed Gibbs free energy of formation for metabolite *j*, corrected for its actual, non-standard metabolite concentration, *c_j_*. R is the molar gas constant, and *T* the absolute temperature. 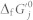 represents the transformed standard Gibbs free energy of formation, which we corrected for ionic strength and pH^21^. See the supplementary materials, section “Calculation of the transformed standard Gibbs free energy of formation” on page S-26 for details and the supplementary materials, file 2 for actual 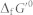 -values.

Eqs. (1) and (2) identify isolated, thermodynamically infeasible reactions based on (measured) metabolite concentrations. However, NET analysis does not only study a reaction in isolation, but rather considers a reaction's feasibility in the context of pathways. NET analysis utilizes the thermodynamic interdependencies between reactions and verifies if a given network structure is consistent with a (measured) metabolome. To this end NET analysis is solved by the linear program (LP) given by^22^
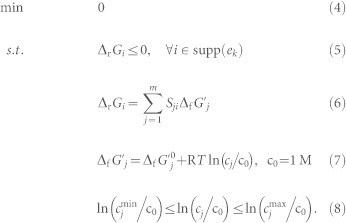
The program above is linear in ln(*c_j_*/c_0_). That is why the limits in Eq. (8) were expressed in terms of logarithms. The LP checks whether all reactions contributing to an EFM, *e_k_*, are simultaneously feasible [Eq. (5)] and consistent with a metabolome within the given error bounds 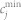 and 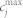, respectively [Eq. (8)]. The remaining equations [Eqs. (6) and (7)] account for mapping the metabolome to the Gibbs free energy surface. Note that in the original NET analysis^20^ Eqs. (4-8) are optimized for Δ_r_*G_i_*, while we are only interested in the feasibility of Eqs. (4-8). Therefore, any (non-optimal) solution suffices, which poses a computationally less challenging problem.

**Figure 1:**
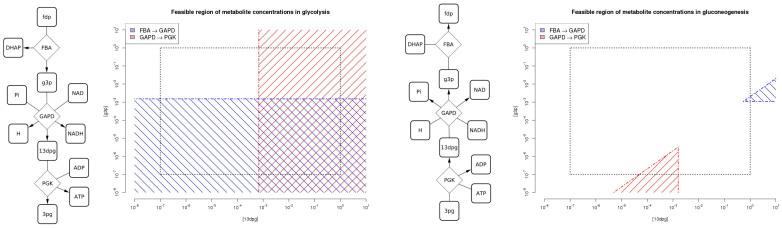
Thermodynamically feasible concentration regions for 1,3-bisphosphoglycerate (13dpg) and glyceraldehyde-3-phosphate (g3p) at glycolysis (left) or gluconeogenesis (right) for *E. coli* when growing on minimal media with glucose. Dashed lines indicate the concentration bounds of the metabolites and chain dotted lines the bound of negative Gibbs energy, i.e. the line where Δ_r_*G*_GAPD_ = 0. Blue areas show regions of negative Gibbs energy for the combination of FBA (fructose-bisphosphate aldolase) and GAPD (glyceraldehyde-3-phosphate dehydrogenase) and red areas for the combination of GAPD and PGK (phosphoglycerate kinase). At glycolysis all three reactions are simultaneously thermodynamically feasible indicated by the overlapping red and blue area. At gluconeogenesis such an overlap within the error bounds of the metabolites cannot be found. To find the feasible regions we analyzed the admissible concentrations of the shared metabolites 13dpg and g3p. The minimum and maximum concentration of g3p as function of 13dpg was calculated so that Δ_r_*G_i_* ≤ 0 held for all reactions.

The basic feature of NET analysis is illustrated in Figure 1. In isolation, each reaction (FBA, GAPD, and PGK) is feasible in both directions. Also the reaction pairs (FBA, GAPD and GAPD, PGK) are feasible in both directions. However, if the reaction triple (FBA, GAPD, PGK) is considered, we find only the forward direction to be consistent with the metabolite concentrations.

**Figure 2:**
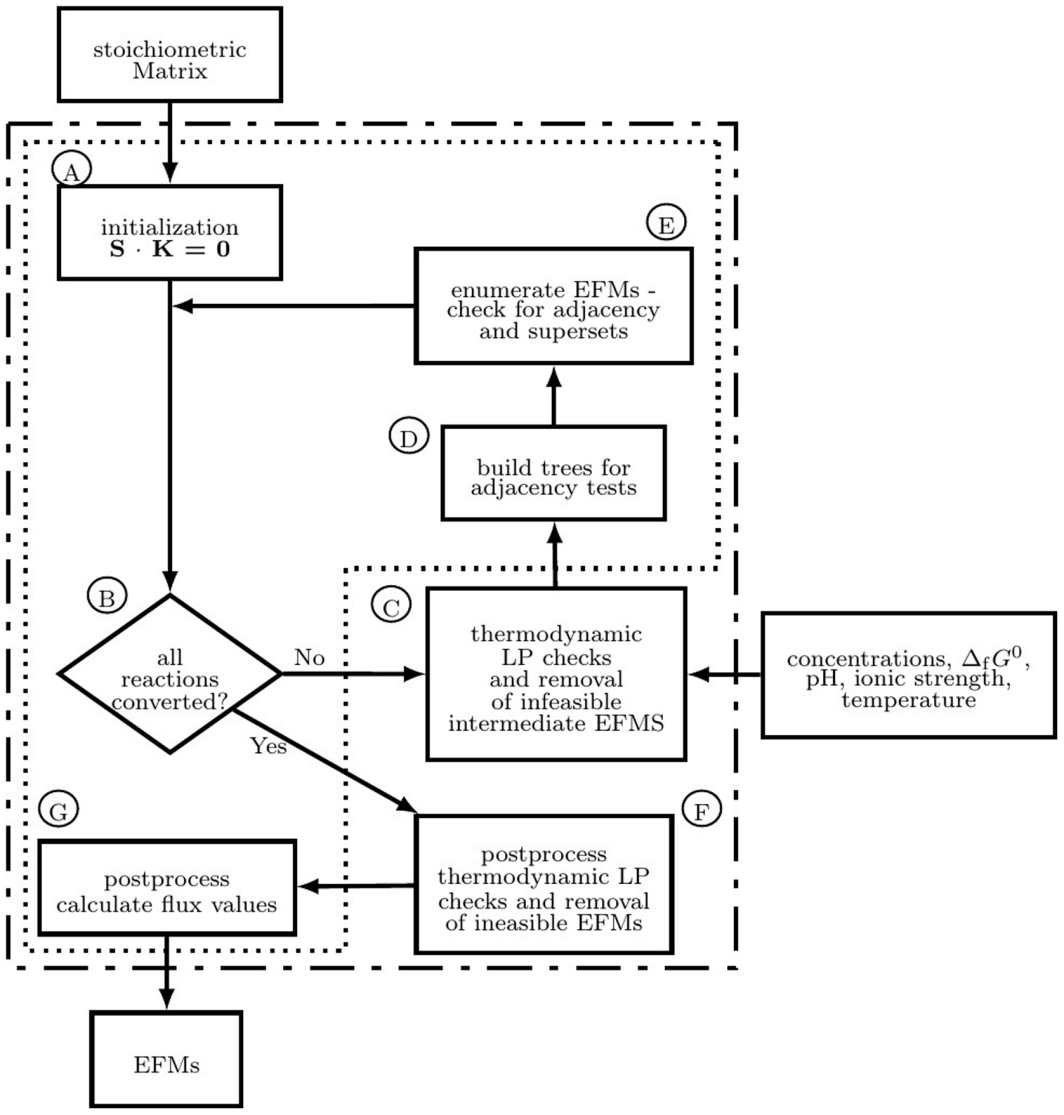
Basic work flow of tEFMA. Dashed lines mark the original *efmtool* and chain dotted lines the integration of NET analysis into tEFMA. (A) In the initialization phase the stoichiometric matrix is compressed and the kernel matrix created. (B) As long as a reaction is not converted from numeric to binary a new iteration is started. (C) Intermediate EFMs with positive or negative values on next numeric position are checked for thermodynamic feasibility, based on given input values. Infeasible EFMs are removed here. (D) Adjacency trees are built with EFM intermediates. (E) New intermediate EFMs are created by combining adjacent EFMs from positive and negative trees. They are added to the list of intermediate EFMs unless they are supersets of other intermediates. (F) In the post-processing phase calculated EFMs are finally checked to be thermodynamic feasible. In the last step (G) *efmtool* removes futile-2-cycles, decompresses EFMs and calculates the flux values resulting in the enumerated set of EFMs. For an example see the supplementary material, section “Proof of safe removal of thermodynamically infeasible EFMs” on page S-15.

In tEFMA every intermediate EFM is checked at the beginning of each iteration against a given metabolome according to Eqs. (4-8) and immediately removed if infeasible. Figure 2 illustrates the basic work flow. For example, in iteration *i* we may find that 18, 41, and 12 intermediate EFMs have positive, zero, and negative flux values in reaction *i*. This gives rise to 18 × 12 = 216 potentially new intermediate EFMs of which only 22 EFMs are actually added to the list of new intermediates as only these pass the (tree-based) adjacency and superset testing of the EFM enumeration procedure. In tEFMA we check the feasibility of the original 18 + 12 intermediate EFMs and remove infeasible EFMs there. Suppose that 8 out of the 18 positive intermediate EFMs are infeasible and can be removed instantly. Rather than 216 potentially new intermediates we now just get 10 × 12 = 120 potentially new intermediates of which only 17 EFMs are actually added to the list of new intermediate EFMs as these pass the (tree-based) adjacency and superset testing. (The numbers for this example were taken from line *i* = 10 in Table S5.) Note that the combination of two intermediate EFMs may create new infeasibilities. If these new intermediates have non-zero flux values in any of the so far unprocessed reactions, they will be checked in a later step of the iteration procedure. In case a new intermediate EFM has only zero flux in the remaining reactions, it will be detected at the end of the iteration phase, where we run a final feasibility check on all remaining EFMs (see Figure 2).

The efficiency of this approach is illustrated in Table S5, Table S6, and Table S7, where we show that the total number of LPs is always smaller than the total number of newly generated intermediate EFMs in the non-thermodynamic EFMA. We found heuristically that it is more efficient to check the feasibility of intermediate EFMs first and then do the tree-based adjacency and superset testing, rather than the other way round (data not shown).

In the remainder we assume that *T* = 310.15 K (37°C).

### Implementation

We implemented tEFMA as an extension of the open source software *efmtool*, which was originally developed by Terzer and Stelling^23^. We added three new Java packages with 21 new Java classes to *efmtool*. The new classes are responsible for reading the additional information, call CPLEX (a powerful commercial solver by IBM, for which academic licenses are available on request) and handle infeasible EFMs. To invoke the new functionality we modified two already existing Java classes and the XML file that handles command line arguments (see the README-file in the accompanying software package^24^ for details). The extended version was compiled by JDK 1.7.11.

### Metabolic reconstructions

We used the *E. coli* core model published by Orth *et al.*^25^. We refer to it as model M1. M1 contained 73 metabolites and 155 irreversible reactions (after splitting each of the 59 reversible reactions into two irreversible forward and backward reactions). The core reconstruction, M1, does not model glycerol uptake, so we added the glycerol uptake pathway from the *E. coli* model *i*JR904^26^. This augmented model is referred as model M2. Specifically, we included glycerol kinase (R GLYK), glycerol-3-phosphate dehydrogenase (R G3PD2, R G3PD5), glycerol transport (R GLYCt) and glycerol exchange (R EX glyc e). The resulting stoichiometric matrix consisted of 76 metabolites and 163 reactions (62 of them were initially reversible). The rank of this matrix was 71. We used M2 to derive three condition specific sub-models, M2-glc, M2-glyc, and M2-ac, to model growth on minimal medium (containing ammonia, oxygen, phosphate, protons, and water) with glucose, glycerol or acetate as the sole carbon source, respectively. In these models all uptake reactions for nutrients which were not included in the growth media, were removed. If a nutrient transport was reversible we only disabled the nutrient's uptake but not its secretion into the extracellular environment.

Except for glutamate and glutamine, neither M1 nor M2 model the biosynthesis of the other amino acids. Thus we augmented M2 by adding the amino acid pathways extracted from the *E. coli* model *i*JE660a^27^. This model is referred to as M3. Its stoichiometric matrix consisted of 178 metabolites and 303 irreversible reactions (94 of them were initially reversible). The rank of this matrix was 171. SBML files for M2 and M3 are available in the supplementary materials. M1 can easily be obtained by removing R GLYK, R G3PD2, R G3PD5, R GLYCt, and R_EX_glyc_e from reconstruction M2.

We summarized the main topological properties of all models in the supplementary material, Table S4.

### Functionality test

We tested tEFMA for specificity, sensitivity and performance. For the thermodynamic feasibility checks we used previously published metabolite concentration data for *E. coli* when grown on glucose, glycerol or acetate^28^. In comparison to published concentration ranges^29^, we used very conservative minimum (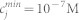) and maximum (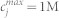) default values for unmeasured metabolites to avoid false identification of infeasible EFMs. The necessary Δ_f_*G*^0^ data were taken from the online version of eQuilibrator^30^. Independently, we performed a conventional EFMA on the same model using *efmtool* and separately tested each EFM for thermodynamic feasibility using NET analysis.

### Stability analysis

We tested the stability of tEFMA against perturbations in the metabolome and the thermodynamic data. We randomly changed all concentrations up to ±5%, ±10%, ±15%, and ±20%. This change was on top of the error bounds given by Ref. 27. That is, all lower and upper bounds (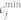 and 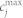, respectively) were independently changed within the intervals given above. Additionally we required that 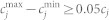, where we used *c_j_* to denote the mean concentration of metabolite *j*. The perturbed concentrations were then used in the tEFMA. The whole procedure was repeated 100 times. Similarly, all 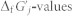 were perturbed by randomly and independently changing each value by up to ±0.3 kJ/mol, ±1 kJ/mol, ±3 kJ/mol, and ±9 kJ/mol. Again, this procedure was repeated 100 times.

## Results

We calculated thermodynamically feasible EFMs in medium scale metabolic models of *E. coli* (models M1 to M3) based on experimental metabolite concentrations measured by Bennett *et al.*^28^. In the smallest reconstruction (model M1), the experimental data accounted for 56% of the model's internal metabolites. All unmeasured metabolites were assumed to be within conservative concentration bounds (see method section for details). 15 out of 155 irreversible reactions in M1 were thermodynamically fully characterized by measurements. 56 reactions were at least partially characterized by experimental data. The overlap between the model M1 and the experimental data is illustrated in the supplementary material, Figure S1.

### Computational, tEFMA identifies thermodynamically feasible EFMs accurately and economically

#### tEFMA removes all infeasible EFMs

**Figure 3:**
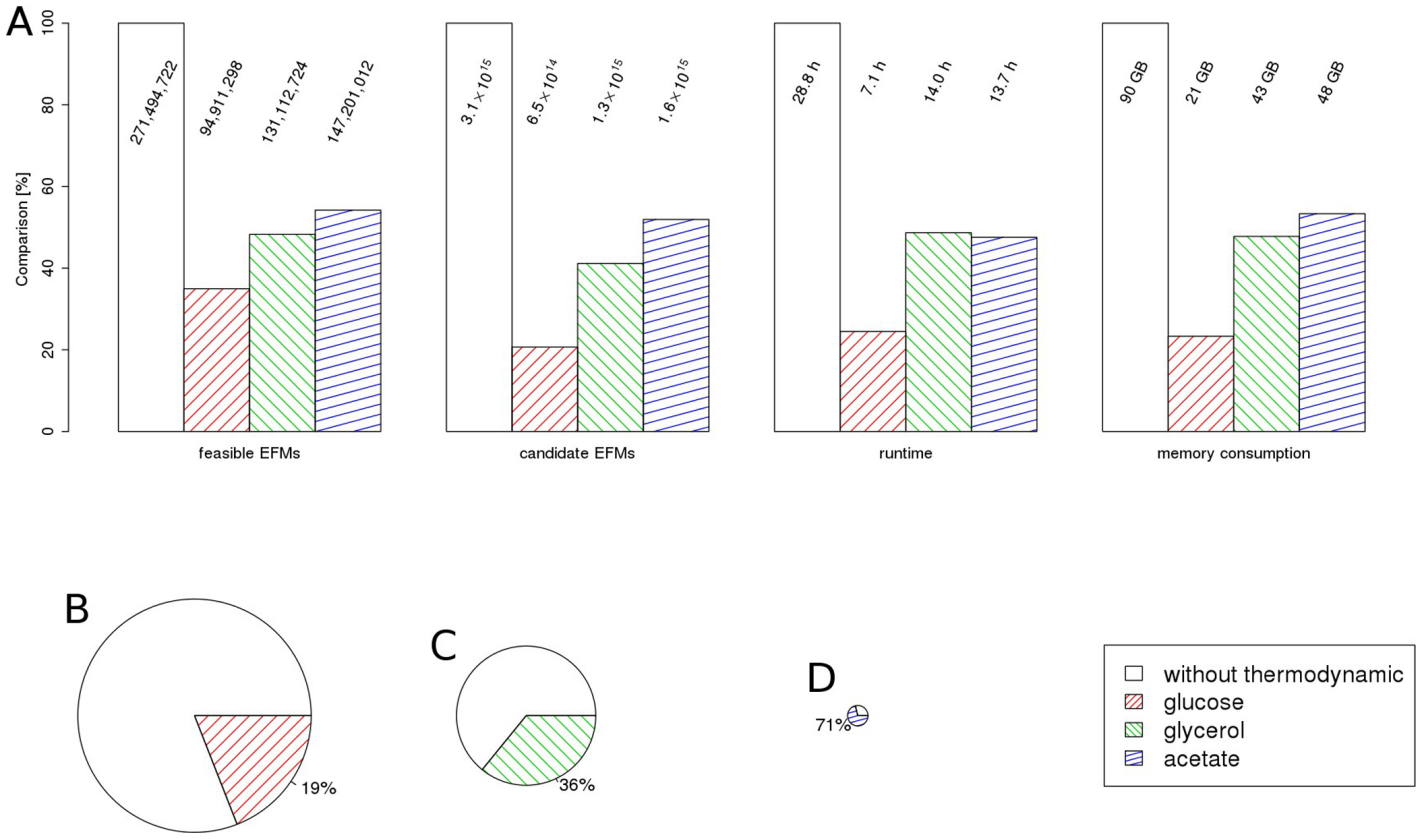
Performance analysis of tEFMA with and without thermodynamic feasibility checks using three different metabolomes. Results of an ordinary EFMA (none) were compared against tEFMA using metabolome data^27^ for growth on minimal medium (MM, contained ammonia, oxygen, phosphate, protons, and water) and glucose (glc + MM), glycerol (glyc + MM), and acetate (ac + MM). The analysis was performed (A) on the *E. coli* model M1 and (B–D) on condition specific model M2, where all inactive uptake reactions were removed. Using glucose (B) 32,374 EFMs out of 169,916 are feasible, whereas for glycerol (C) 21,642 out of 60,495 and for acetate (D) 925 out of 1,299 EFMs are thermodynamically feasible. In panel A numbers on the top indicate the absolute values. In panel B to C the circle areas are scaled as to represent the total number of topological feasible EFMs in the models.

We compared tEFMA against an ordinary EFMA followed by NET analysis. The sets of thermodynamically feasible EFMs were identical in both analyses. Figure 3 illustrates a comparison between an EFMA and a tEFMA.

For growth on glucose about one third of all EFMs were thermodynamically feasible. The reduction in the number of feasible EFMs is highly condition specific as on glycerol and acetate the numbers of feasible EFMs are roughly cut in half. These comparisons were based on the full metabolic model, M1, without any other adaptations, i.e. also unused uptake reactions were subject to the analysis. If all unused uptake reactions were removed from the models, then the changes in the number of feasible EFMs was even more pronounced.

On glucose minimal medium only 19% of the EFMs were thermodynamically feasible, while on acetate minimal media 76% were feasible (using model M2). However, in the case of glucose 19% corresponded to more than 30,000 feasible EFMs, while on acetate roughly 900 EFMs remained feasible. Thus, growth on glucose still opened dramatically more metabolic possibilities (counted by the number of feasible EFMs) than growth on any other carbon source.

#### tEFMA is stable against fluctuations in the metabolome and the thermodynamic data

We verified the stability of the feasible EFMs by randomly perturbing the metabolite concentrations (see methods section for details). For all tested perturbations (0, …, ±20%) the median number of feasible EFMs remained constant (see the supplementary material, Figure S3). Moreover, all EFMs identified to be feasible without perturbations where re-identified to be feasible in the perturbed runs as well (except for statistical outliers in the case of glycerol and acetate growth at ±20%). Note, that the perturbations were added on top of the experimental error (see the supplementary material, Figure S4 for details.)

We repeated the analysis to also check tEFMA against variations in Δ_f_*G*′-values (see methods section for details). Up to a perturbation magnitude of 1 kJ/mol our results stayed essentially constant (see the supplementary material, Figure S2 and supplementary file 3), i.e. in all these cases we found the same set of EFMs to be thermodynamically feasible. For stronger perturbations large deviations were found.

#### tEFMA strongly reduces runtime and memory usage

Our novel software extended *efmtool* originally developed by Terzer and Stelling^23^, which uses a variant of the double description method (DDM)^31^ to enumerate EFMs. The method requires to repeatedly solve intermediate EFM enumeration problems, which gives rise to a huge number of intermediate EFMs. Although most of these intermediate EFMs will eventually be rejected, they have to be readily available throughout the calculation. This places high demands on a computer's storage capacity [specifically on the size of the random access memory (RAM)]. Figure 3 illustrates the decrease in the number of feasible EFMs for an ordinary EFMA and a tEFMA. The decrease is even stronger in the total number of adjacency candidates, which relaxed the hardware requirements for tEFMA. In fact, RAM consumption in tEFMA is at least cut in halve but can in optimal cases shrink to only 25% compared to the RAM consumption of an ordinary EFMA. Similarly, tEFMA also reduces the runtime of the algorithm and needs only 25% in the best case and 49% in the worst case as compared to an ordinary EFMA (see Figure 3).

### Biological, tEFMA identifies known infeasible pathways

For the following biological interpretation of the calculated EFMs and infeasible pattern we used the model M2.

It is textbook knowledge that under aerobic conditions malate dehydrogenase (Mdh) oxidizes malate to generate oxaloacetate as part of the tricarboxylic acid cycle. tEFMA correctly identified the reverse reaction to be infeasible. Similarly acetaldehyde dehydrogenase (AdhE), which catalyzes the reduction of acetyl coenzyme A to acetaldehyde, was identified to be infeasible under the three tested growth conditions in accordance with well established knowledge. Both conclusions could have been made without the help of tEFMA, as evaluating Eqs. (2) and (3) for Mdh and AdhE unambiguously identified the reactions' directions without considering the network structure of metabolism.

#### tEFMA correctly distinguished between glycolysis and gluconeogenesis

tEFMA correctly classified gluconeogenesis to be infeasible in *E. coli* grown on glucose. The latter could not have been concluded without a NET analysis or tEFMA. For example, within the error bounds of the measured metabolite concentrations the reactions phosphoglycerate kinase (Pgk), glyceraldehyde 3-phosphate dehydrogenase (Gapd), and fructose bisphosphate aldolase (Fba) were found to be reversible if analyzed individually. Only together tEFMA identified them to be infeasible in direction of gluconeogenesis (see Figure 1). The lower part of gluconeogenesis (from pyruvate to glyceraldehyde-3-phosphate) was also predicted to be infeasible for growth on glycerol while feasible for growth on acetate. Interestingly, gluconeogenesis via succinyl coenzyme A synthetase (SucCD) was inaccessible in the latter (see below and Table S3 for further details). Note that Pgk, Gapd, and Fba build a linear, consecutive chain of reactions. In general, however, tEFMA is able to identify thermodynamic inconsistencies between non-consecutive reactions, too (see Table S1 to Table S3).

#### tEFMA correctly predicted the inactivity of glutamate dehydrogenase (GDH) during growth on glucose

**Figure 4:**
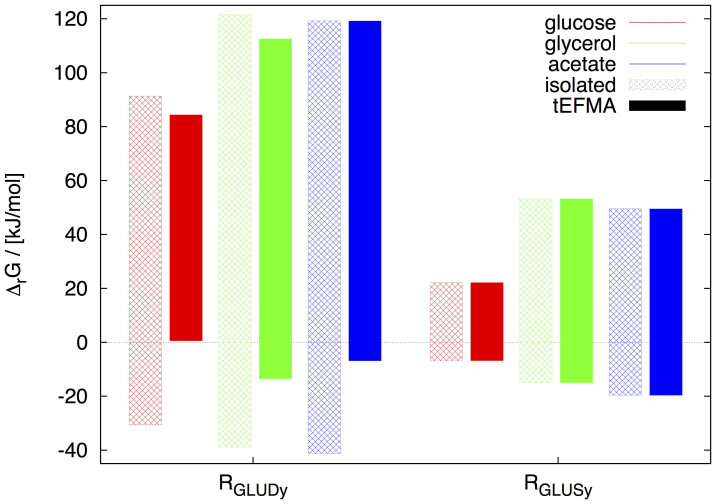
Minimum lower and maximum upper bounds of Δ_r_*G* for the reactions glutamate dehydrogenase (R_GLUDy_) and glutamate synthase (R_GLUSy_) for various conditions in model M2. For each single EFM, which was enumerated by efmtool (without the tEFMA extension), the minimum and maximum Δ_r_*G* of both reactions were calculated in isolation (open pattern) and within a NET analysis (solid pattern). Note, that only negative Δ_r_*G* ranges are thermodynamically feasible. Therefore R_GLUDy_ is never feasible, when grown on glucose and analysed by a NET method (red solid pattern).

Two pathways for glutamate synthesis are known in *E. coli*. GDH catalyzes the reductive amination of *α*-ketoglutarate to form glutamate. Alternatively the glutamine oxoglutarate aminotransferase (GOGAT) pathway produces glutamate in two steps: (i) glutamate is used to produce glutamine by the energy dependent glutamine synthase and (ii) the amide group is then transferred reductively from glutamine to *α*-ketoglutarate to form glutamate. Both pathways were identified in an ordinary EFMA and produce 1 mole of glutamate net. For growth on glucose, however, tEFMA identified inconsistencies between GDH and the lower part of the glycolysis as well as between reactions GDH and aconitate hydratase (ACONTb). We found that on glucose no thermodynamically feasible EFM was supported by an active GDH (see Figure 4). This is consistent with experimental evidence that under glucose saturated conditions the alternative GOGAT pathway is active, and not GDH^32^. On the other hand, we identified thermodynamically feasible, GDH supported EFMs when *E. coli* was grown on acetate or glycerol. Again, this is consistent with experiments, as GDH, but not GOGAT, is energy neutral and therefore favored under energy-stressed conditions^33^. Our analysis revealed that under glucose saturated conditions both reactions are potential thermodynamic bottlenecks as they operate close to Δ_r_*G* = 0^29^. However, GDH was found to be more sensitive than glutamate synthase (see Figure 4). Note that in this analysis it is essential to consider the network structure of metabolism. Within tEFMs GDH is inactive, but by analyzing GDH and glutamate synthase in isolation the inactivity of GDH cannot be determined. In fact a naive interpretation might lead to the erroneous assumption that glutamate synthase rather than GDH is a thermodynamic bottleneck (see Figure 4 for an illustration).

#### tEFMA did not predict false positives

For a given metabolome tEFMA found combinations of reactions that could not operate simultaneously (see Table S1–Table S3). We were able to understand all of these combinations of reactions in terms of the (infeasible) pathways described above. In the three test cases tEFMA did not erroneously identify a thermodynamically feasible pathway to be infeasible.

### tEFMA is scaleable to larger systems

We repeated a tEFMA using the same experimental data as above together with a more detailed *E. coli* reconstruction, M3. This model did not only contain the core carbon metabolism but was augmented with biosynthesis routes for amino acid production. Compared to its parent model, M3 contained roughly twice as many reactions and also twice as many internal metabolites. The overlap between this model and the experimental data is shown in Figure S1. In this model tEFMA identified 1,197,839 thermodynamically feasible EFMs, 37 times more feasible EFMs than in the smaller parent model M1.

In addition, tEFMA identified 15 infeasible flux patterns, i.e. reactions which together must not carry flux (see the supplementary material, Table S9 for a listing). The six infeasible flux patterns detected earlier, in the smaller parent model M2, were also found now in the larger reconstruction. The remaining infeasible patterns could not have been detected in the smaller parent model M2, as they all contained reactions which were unique to the larger M3-model.

## Discussion

We developed and applied tEFMA to study the metabolic capabilities of *E. coli*. tEFMA integrates experimentally determined metabolomes into an ordinary EFMA to avoid the calculation of thermodynamically infeasible EFMs. Recently this strategy was successfully applied to analyze the metabolic capabilities in yeast grown on glucose^22^. The authors first constrained the metabolic network as tightly as possible and then performed an ordinary EFMA followed by a NET analysis on the EFMs. In contrast to this sequential approach, tEFMA efficiently performs both analyses simultaneously, yielding in huge computational savings. Harvesting these savings is the major achievement of this work.

We exploited the fact that any combination of infeasible EFMs with other (in)feasible EFMs is again infeasible^22^ and can be removed from the analysis without impacting biologically relevant EFMs. By doing so, we tackled the major bottleneck in the DDM^31^, i.e., the exploding number of (intermediate) EFMs during the calculation^15^.

Currently DDM is the most common approach for calculating EFMs^9^,^34^. It solves the enumeration problem iteratively by adding one constraint at a time and (re-)enumerating the problem. This is done by a pairwise combination of positive and negative intermediate EFMs. Of the huge number of potential candidates only those intermediate EFMs are used to generate offspring, if they are adjacent. Each newly created intermediate EFM undergoes a superset test which prevents further processing of a new intermediate EFM if it is a superset of any already existing intermediate EFM. Performing the adjacent and superset test, as well, as creating and maintaining this large list of intermediate EFMs is computationally expensive. While Terzer and Stelling^23^ efficiently perform adjacency and superset checks using binary bit pattern trees, we also shorten the overall length of of intermediate EFMs. By running a NET analysis at every iteration on all (positive and negative) intermediate EFMs we identify infeasible ones and remove them at the moment of birth even before the bit pattern trees are created and adjacency tests are performed. Therefore, infeasible EFMs were unable to proliferate and to inflate the list of (intermediate) EFMs with irrelevant offspring. This dramatically reduced the memory requirements. In fact, if we only used the measured glucose metabolome and the M1-model for tEFMA, a current, high-end personal computer (typically 32GB RAM) would suffice to perform the analysis in a single working day and eliminate the need for a dedicated high performance computing environment. Conversely, tEFMA allowed us to analyse larger systems, which were inaccessible to an ordinary EFMA on our computer infrastructure.

To curb the explosion of the number of (intermediate) EFMs, we solved many LPs to determine their feasibility. In our application LPs are uncritical in terms of memory consumption. Overall we saved memory by removing infeasible EFMs at the price of an increased computational load to evaluate the LPs. Fewer (intermediate) EFMs meant a shorter list of (intermediate) EFMs, too. This reduced the time to perform the adjacency and superset tests on the EFMs. In the tested cases, the overall runtime decreased at least by 50%. Note that the scaling and efficiency of the DDM critically depends on the order in which constraints are processed^31^,^34^. This remains also true for tEFMA (data not shown).

It is known that out of all EFMs in large networks few are physiologically significant^35^. Ideally only those will also be calculated. tEFMA (partly) reaches this aim. By adding constraints derived from metabolomics data we reduced the solution space, leading to a substantial reduction in the number of EFMs without loosing any biologically relevant EFMs. However, tEFMA only identifies thermodynamically infeasible EFMs. For instance, during growth under high glucose conditions the glyoxylate shunt is inactive due to regulatory interactions. This is not detected by tEFMA. Therefore tEFMA alone does not allow for an EFMA of a (large) genome scale model. In fact, we were unable to complete a tEFMA on a current genome scale model of *E. coli* on our computer infrastructure. More (omics-)data, like regulatory constraints^15^, need to be included to tighten the solution space and get rid of irrelevant EFMs. Recently, gene expression data was used to calculate a small subset of characteristic EFMs^36^ in genome-scale networks. In contrast to their method, however, tEFMA is comprehensive and builds on first principles, rather than statistical heuristics. Nevertheless a combination of their method with tEFMA is required to fully enumerate EFMs in genome-scale models, which is the scope of further work.

Although tEFMA utilizes an optimization principle to fit the metabolic profile, it still retains the ability to unbiasedly characterize all metabolic capabilities of an organism. However, tEFMA cannot predict individual metabolic fluxes. In fact, even the combination of two thermodynamically feasible EFMs might result in an infeasible flux distribution^22^. This is in contrast to thermodynamic-based metabolic flux analysis, where an optimization principle is used to determine a particular thermodynamically feasible flux distribution^29^,^37^,^38^. Predicting intracellular flux distribution from an EFM-spectrum is an active field of research^39^. In fact, metabolite data have increasingly been utilized together with EFMA in order to gain more reliable flux estimates^40^,^41^,^42^,^43^. However, in all these studies an EFMA was carried out first (on a small-scale metabolic model), while the thermodynamic feasibility was only checked *a posteriori*. tEFMA will aid such studies in providing better computational performance and allowing larger systems to be analyzed.

The success of tEFMA is dependent on the availability of a measured metabolome. Measurement errors in the concentrations were taken into account, and tEFMA was found to be robust against further perturbations. More critical for tEFMA is the requirement for accurate data on the Gibbs free energy of formation, Δ_f_*G*, for each metabolite. Our analysis showed that an error in Δ_f_*G* of up to 1 kJ/mol did not cause alterations. Such accuracy is achievable with current (reactant contribution) methods for the estimation of the Gibbs energy^44^. However, these data cover less then one tenth of the reactions in a typical genome scale model. Yet they are sufficient for the kind of medium-scale models accessible to tEFMA. Thus even if only a small fraction of the metabolome were available, tEFMA will still provide a computational advantage. Moreover, missing data do not lead to the identification of false positives. Uncharacterized reactions can simply be omitted in NET analysis. Consequently some thermodynamically infeasible EFMs will not be detected and the overall efficiency of the algorithm will be reduced.

tEFMA is inherently condition specific and in principle has to be repeated upon any change in the environment. In practice, however, that might not be necessary as Ishii *et al.*^45^ observed that metabolite levels were remarkably stable against perturbations.

tEFMA's condition specificity is in strong contrast to the approach taken by Hunt *et al.*^9^. Those authors pinned their approach on massive parallelization by recursively splitting the network in appropriately selected subnetworks and performing an EFMA there. As the authors did not utilize any additional information, their enumeration is complete and has to be run only once. However,they found close to two billion EFMs in a large-scale model of *P. tricornutum*^9^. The sheer scale makes an interpretation of the EFMs difficult and computationally challenging. Extrapolating our results onto their model, we expect that many EFMs will be infeasible and therefore biologically irrelevant. This could be easily checked by running a NET analysis on their set of EFMs, if experimental data were available. As both approaches are DDM based, it should be possible to integrate tEFMA into the approach of Hunt *et al.*^9^.

tEFMA retains the ability to allow for a fully unbiased analysis of metabolism. In fact, the predicted inactivity of GDH under growth on glucose was completely derived from first principles. This allows to draw very general statements of biological relevance without relying on optimality criteria or particular flux distributions. The inactivity of GDH for instance, allows glutamate synthesis only via the ATP consuming GOGAT pathway. The increased energy demand for glutamate synthesis might cause problems during recombinant protein production, which induces additional energy requirements in the host. Thus by activating GDH rather than GOGAT the metabolic burden is reduced.

Currently an assumption-free tEFMA can only be performed on prokaryotes. tEFMA on eukaryotes would require compartment specific concentration data. A theory to describe the thermodynamics of inter-compartmental transport is available^46^, yet current experimental methods do not allow for a compartment specific resolution of the metabolome. In order to apply tEFMA also to compartmentalised organisms *ad hoc* assumptions are required to estimate the missing compartment specific concentration data^22^.

In summary, we developed tEFMA, a tool that presents an important step forward to the analysis of genome-scale metabolic networks. tEFMA integrates NET analysis into EFMA and succeeds in calculating only EFMs, that are thermodynamically consistent with a given metabolome. By doing so, it dramatically reduces the hardware requirements for such an analysis to be carried out and paves the way to enumerate EFMs in large-scale metabolic networks. This is possible as the calculated set of EFMs is reduced by the large number of thermodynamically infeasible EFMs. To show the accuracy of the tool we presented the correct identification of several infeasible pathways without making wrong predictions. Furthermore, we pointed out that tEFMA correctly distinguishes between the GDH and GOGAT pathways to produce glutamate. Additionally, we verified that the patterns, and therefore pathways, which were found to be infeasible in the smaller model remained infeasible in the larger model.

## Author Contributions

M.P.G., C.J. and J.Z. conceived and designed the study; M.P.G. and C.J. developed the software; M.P.G. and J.Z. developed the models; M.P.G. run the experiments; M.P.G. and D.E.R. contributed analysis tools and performed the analysis; M.P.G., D.M., C.J. and J.Z. wrote the manuscript.

## Supplementary Material

Supplementary Information Supplementary file 1

Supplementary Information Supplementary file 2

Supplementary Information Supplementary file 3
