# Supplementary material for: Metabolomics integrated elementary flux mode analysis in large metabolic networks
Source: Sci Rep. 2015 Mar 10;5:8930. doi: 10.1038/srep08930 (PMC4354105; doi:10.1038/srep08930)
Supplement: Supplementary Information — Supplementary file 1 [file srep08930-s1.pdf]

Supplementary Material: Metabolomics integrated elementary flux mode analysis in large metabolic networks

Matthias P. Gerstl, David E. Ruckerbauer, Diethard Mattanovich, Christian Jungreuthmayer & Jürgen Zanghellini

S1–S3 show infeasible patterns for different cultivation media. Diagrams were created with Cytoscape [1] according to the *E. coli* core model [2] with additional glycerol exchange (model M2). Metabolites are drawn as squares and reactions as diamonds. Reactions of the infeasible pattern are marked red. Directions of the reactions are drawn according to the model, except for those that are part of a pattern, where only the infeasible direction is plotted. Infeasibility patterns are also listed in supplementary Table **S9**.

Table **S1**: Minimal infeasible patterns in *E. coli* aerobically growing on glucose.

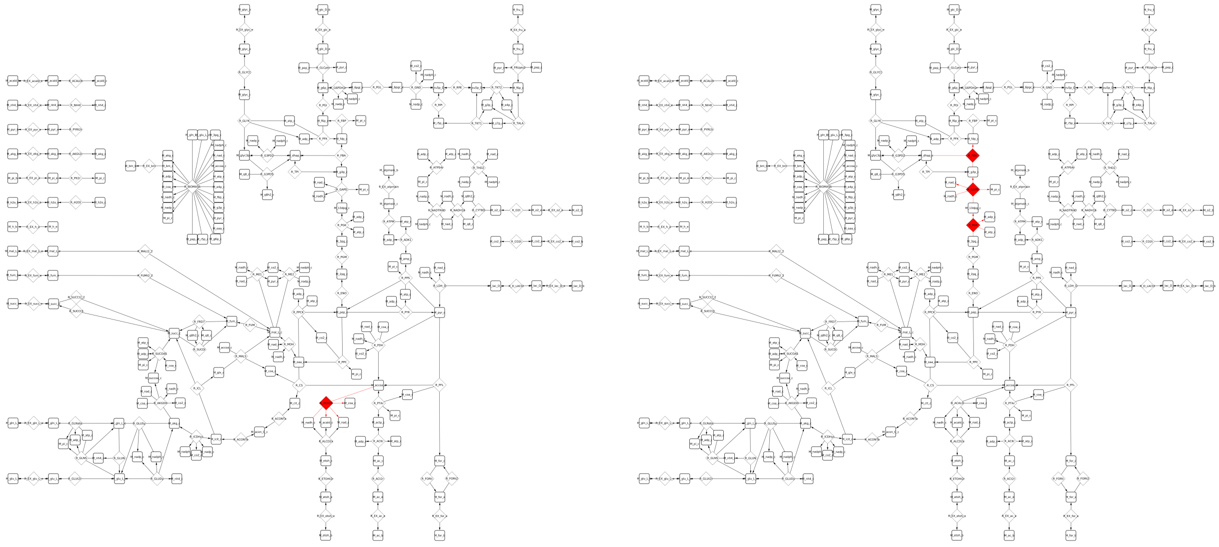

Table **S1** continues on next page.

Table **S1** continues from previous page.

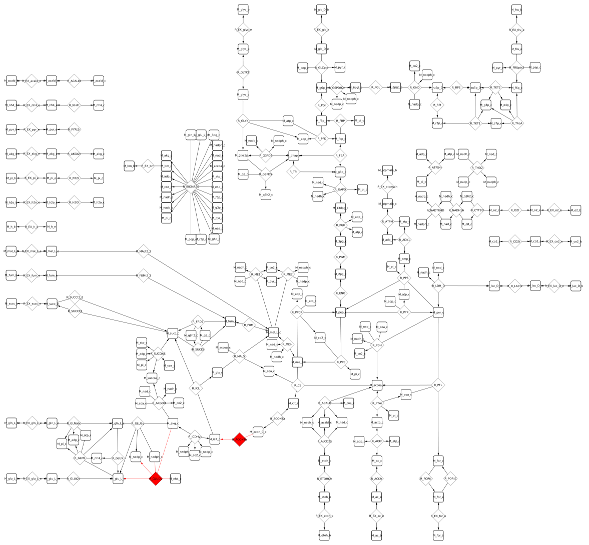

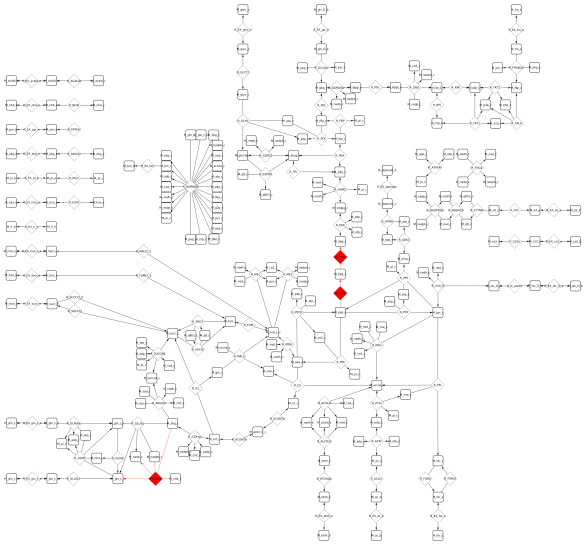

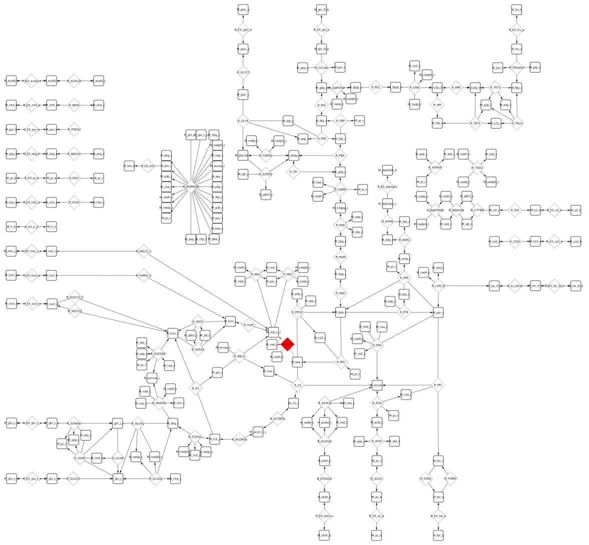

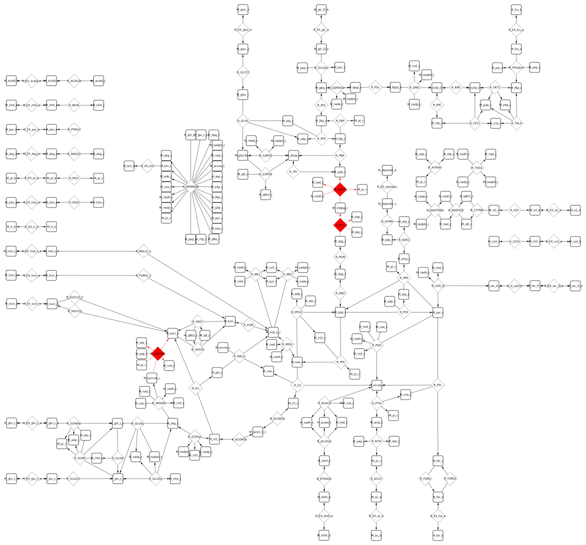

Table **S1** ends.

Table **S2**: Minimal infeasible patterns in *E. coli* aerobically growing on glycerol.

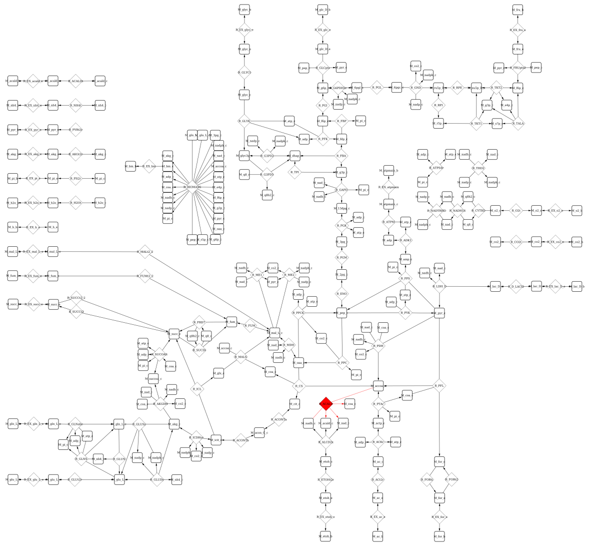

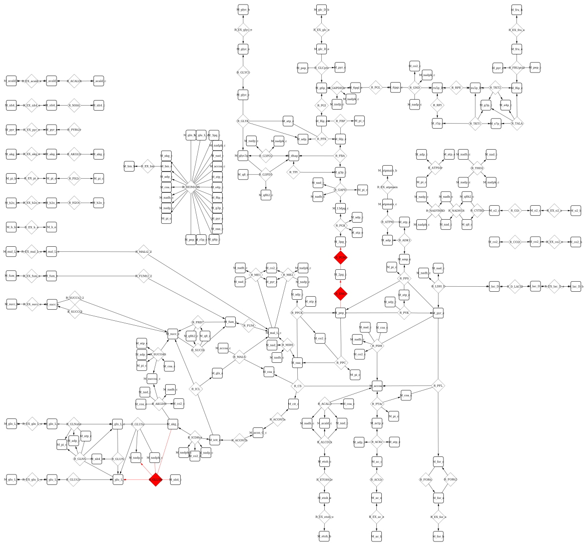

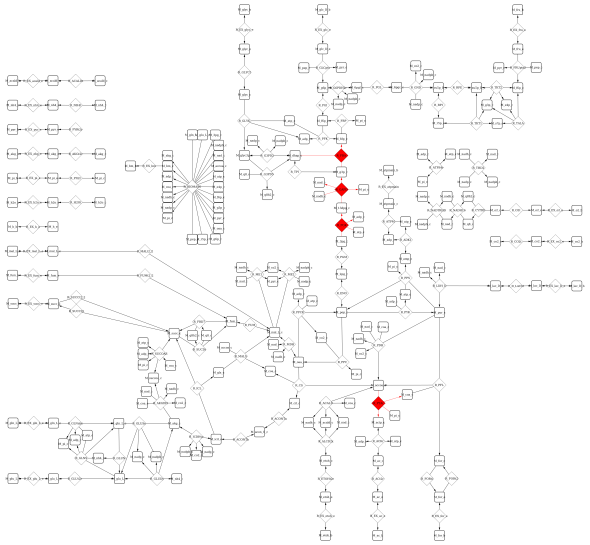

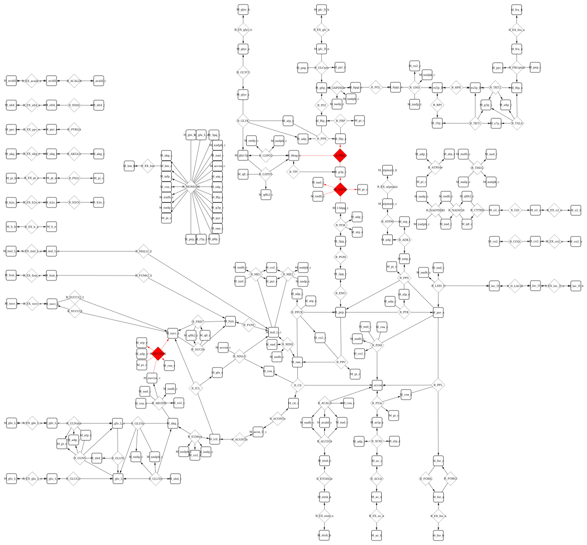

Table **S2** continues on next page.

Table **S2** continues from previous page.

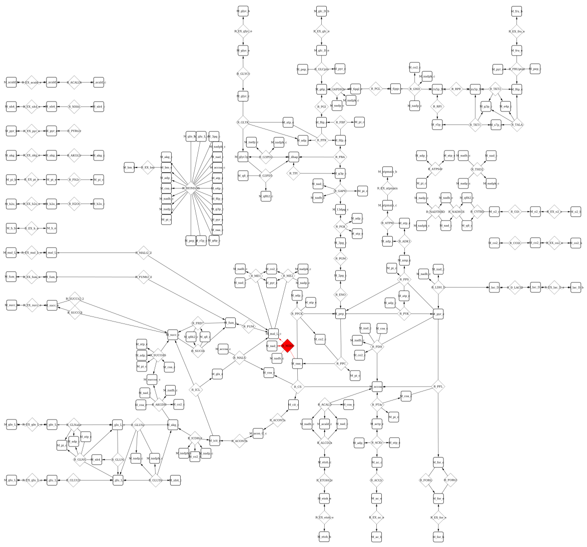

Table **S2** ends.

Table **S3**: Minimal infeasible patterns in *E. coli* aerobically growing on acetate.

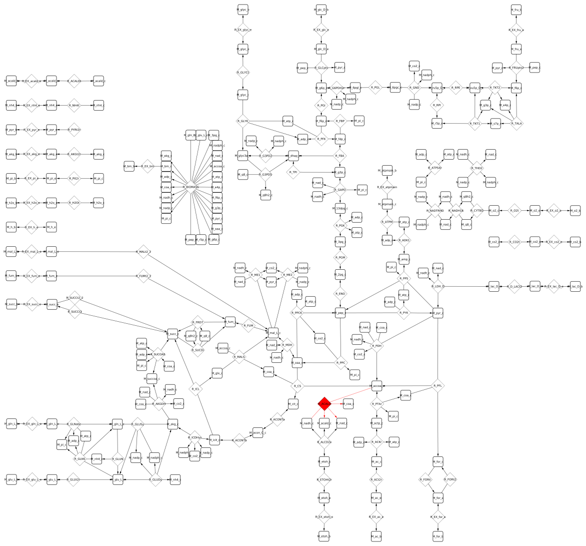

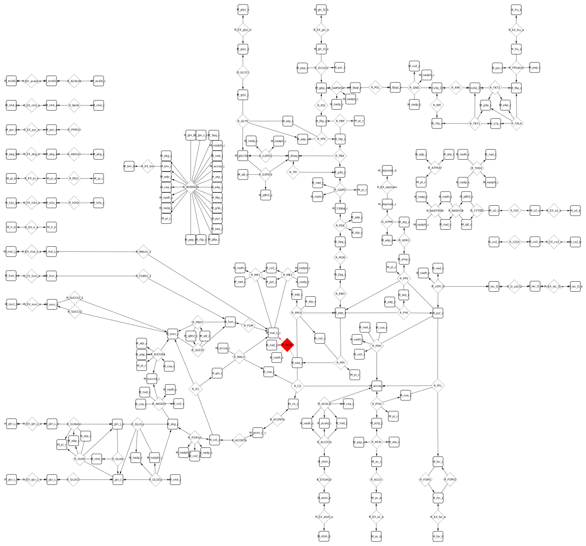

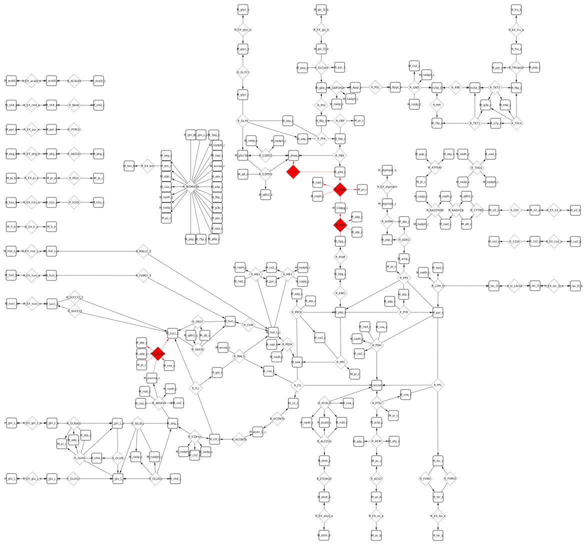

Table **S3** ends.

Table **S4**: Topological properties of used *E. coli* models.

	reactions			
model	irreversible	reversible	metabolites	rank
M1	37	59	73	68
M2	39	62	76	71
M2-glc	53	48	76	71
M2-glyc	53	48	76	71
M2-ac	53	48	76	71
M3	115	94	178	171

Table **S5**: Performance comparison of model M2 grown on glucose. +, 0, - show the number of (feasible) intermediate EFMs (iEFM), having a positive, zero or negative values at reaction (iteration step) i. tEFMA checks the feasibility of all + and - iEFMs. (The total number of checks per iterations is listed in column "LP"). During post-processing (PP) all enumerated (i)EFMs are checked in tEFMA. The columns "cand." and "new" iEFM, list the number of potentially new iEFMs and the actual number of new iEFMs found in iteration i. The column "diff" lists the difference in the number of new iEFMs for both methods. Bold values in the table indicate infeasible iEFMs were detected.

	**tEFMA**									**EFMA**					
i	i**EFM**			**LP**	**feasible** i**EFM**			i**EFM**		i**EFM**			i**EFM**		**diff**
	**+**	**0**	**-**		**+**	**0**	**-**	**cand.**	**new**	**+**	**0**	**-**	**cand.**	**new**	
0	1	39	2	3	1	39	2	2	2	1	39	2	2	2	0
1	3	39	0	3	3	39	0	0	0	3	39	0	0	0	0
2	2	39	1	3	2	39	1	2	2	2	39	1	2	2	0
3	2	40	1	3	2	40	1	2	2	2	40	1	2	2	0
4	3	40	1	4	3	40	1	3	2	3	40	1	3	2	0
5	4	40	1	5	4	40	1	4	2	4	40	1	4	2	0
6	2	39	5	7	2	39	5	10	10	2	39	5	10	10	0
7	10	40	1	11	10	40	1	10	10	10	40	1	10	10	0
8	1	39	20	21	1	39	20	20	20	1	39	20	20	20	0
9	3	40	17	20	3	40	17	51	28	3	40	17	51	28	0
10	18	41	12	30	**10**	41	12	120	17	18	41	12	216	22	5
11	14	48	6	20	14	48	6	84	6	18	53	10	180	10	4
12	16	40	12	28	16	40	**7**	112	13	22	40	19	418	34	21
13	29	39	1	30	29	39	1	29	28	55	40	1	55	54	26
14	2	66	28	30	2	66	28	56	2	2	93	54	108	2	0
15	21	37	12	33	**17**	37	**10**	170	82	46	37	14	644	212	130
16	5	34	97	102	5	34	**86**	430	77	5	34	256	1,280	139	62
17	1	35	80	81	1	35	80	80	80	1	36	141	141	141	61
18	40	26	50	90	40	26	50	2,000	427	64	26	88	5,632	746	319
19	101	211	181	282	101	211	181	18,281	239	135	311	390	52,650	464	225
20	451	36	64	515	451	36	64	28,864	869	767	36	107	82,069	1,524	655
21	93	130	1,133	1,226	**90**	130	**693**	62,370	970	100	131	2,096	209,600	1,778	808
22	1,139	49	2	1,141	**1,052**	49	**1**	1,052	3	1,906	58	45	85,770	710	707
23	315	65	724	1,039	**309**	65	**723**	223,407	1,603	702	135	1,837	1,289,574	6,498	4,895
24	456	617	904	1,360	456	617	904	412,224	788	3,377	1,828	2,130	7,193,010	3,678	2,890
25	1,527	265	69	1,596	1,527	265	69	105,363	564	8,260	502	121	999,460	1,550	986
26	1,085	282	989	2,074	1,085	282	**983**	1,066,555	5,188	4,193	441	5,678	23,807,854	26,025	20,837
27	2,833	124	3,598	6,431	2,833	124	3,598	10,193,134	29,433	10,949	168	19,542	213,965,358	159,271	129,838
PP	–	–	–	32,390	–	–	–	–	–	–	–	–	–	–	–
$\sum$				48,578				12,114,435	40,467				247,694,123	202,936	162,469

Table **S6**: Performance comparison of model M2 grown on glycerol. +, 0, - show the number of (feasible) intermediate EFMs (iEFM), having a positive, zero or negative values at reaction (iteration step) i. tEFMA checks the feasibility of all + and - iEFMs. (The total number of checks per iterations is listed in column “LP”). During post-processing (PP) all enumerated (i)EFMs are checked in tEFMA. The columns “cand.” and “new” iEFM, list the number of potentially new iEFMs and the actual number of new iEFMs found in iteration i. The column “diff” lists the difference in the number of new iEFMs for both methods. Bold values in the table indicate infeasible iEFMs were detected.

	**tEFMA**									**EFMA**					
i	**iEFM**			**LP**	**feasible iEFM**			**iEFM**		**iEFM**			**iEFM**		**diff**
	**+**	**0**	**-**		**+**	**0**	**-**	**cand.**	**new**	**+**	**0**	**-**	**cand.**	**new**	
0	2	40	1	3	2	40	1	2	2	2	40	1	2	2	0
1	1	41	2	3	1	41	2	2	2	1	41	2	2	2	0
2	1	41	2	3	1	41	2	2	2	1	41	2	2	2	0
3	3	41	0	3	3	41	0	0	0	3	41	0	0	0	0
4	2	41	1	3	2	41	1	2	2	2	41	1	2	2	0
5	2	41	2	4	2	41	2	4	3	2	41	2	4	3	0
6	3	41	2	5	3	41	2	6	3	3	41	2	6	3	0
7	1	41	5	6	1	41	5	5	5	1	41	5	5	5	0
8	6	40	1	7	6	40	1	6	6	6	40	1	6	6	0
9	1	39	12	13	1	39	12	12	12	1	39	12	12	12	0
10	13	38	1	14	13	38	1	13	12	13	38	1	13	12	0
11	2	49	12	14	2	49	12	24	2	2	49	12	24	2	0
12	1	34	18	19	1	34	18	18	18	1	34	18	18	18	0
13	10	38	5	15	10	38	5	50	42	10	38	5	50	42	0
14	55	34	1	56	55	34	1	55	16	55	34	1	55	16	0
15	55	41	9	64	55	41	9	495	17	55	41	9	495	17	0
16	18	41	54	72	18	41	54	972	62	18	41	54	972	62	0
17	55	41	25	80	55	41	25	1,375	33	55	41	25	1,375	33	0
18	46	34	49	95	**33**	34	49	1,617	157	46	34	49	2,254	218	61
19	156	46	22	178	156	46	22	3,432	98	217	47	34	7,378	122	24
20	21	43	236	257	21	43	236	4,956	1,137	22	50	314	6,908	1,343	206
21	420	28	753	1,173	420	28	**708**	297,360	5,346	517	30	868	448,756	6,604	1,258
22	1,333	1,917	2,544	3,877	1,333	1,917	**2,534**	3,377,822	3,074	1,641	2,198	3,312	5,434,992	3,954	880
23	5,706	348	270	5,976	5,706	348	270	1,540,620	4,707	6,822	620	351	2,394,522	5,732	1,025
24	4,971	342	5,448	10,419	4,971	342	5,448	27,082,008	29,120	5,665	419	7,090	40,164,850	36,386	7,266
25	33,531	161	741	34,272	**33,530**	161	741	24,845,730	14,225	41,313	192	965	39,867,045	17,733	3,508
26	2,216	605	45,095	47,311	**1,880**	605	**11,946**	22,458,480	13,538	2,523	710	56,005	141,300,615	22,038	8,500
27	7,157	714	8,152	15,309	**6,191**	714	**7,295**	45,163,345	21,729	11,634	1,250	12,387	144,110,358	42,902	21,173
28	4,022	5,002	19,610	23,632	4,022	5,002	**19,570**	78,710,540	12,697	15,754	8,594	31,438	495,274,252	36,165	23,468
pp	-	-	-	21,721	-	-	-	-	-	-	-	-	-	-	
$\sum$				164,594				203,488,953	106,067				869,014,973	173,436	67,369

Table **S7**: Performance comparison of model M2 grown on acetate. +, 0, - show the number of (feasible) intermediate EFMs (iEFM), having a positive, zero or negative values at reaction (iteration step) i. tEFMA checks the feasibility of all + and - iEFMs. (The total number of checks per iterations is listed in column "LP"). During post-processing (PP) all enumerated (i)EFMs are checked in tEFMA. The columns "cand." and "new" iEFM, list the number of potentially new iEFMs and the actual number of new iEFMs found in iteration i. The column "diff" lists the difference in the number of new iEFMs for both methods. Bold values in the table indicate infeasible iEFMs were detected.

	**tEFMA**									**EFMA**					
i	***i*EFM**			**LP**	**feasible *i*EFM**			***i*EFM**		***i*EFM**			***i*EFM**		**diff**
	**+**	**0**	**-**		**+**	**0**	**-**	**cand.**	**new**	**+**	**0**	**-**	**cand.**	**new**	
0	2	38	1	3	2	38	1	2	2	2	38	1	2	2	0
1	1	39	2	3	1	39	2	2	2	1	39	2	2	2	0
2	1	39	2	3	1	39	2	2	2	1	39	2	2	2	0
3	1	39	2	3	1	39	2	2	2	1	39	2	2	2	0
4	2	39	1	3	2	39	1	2	2	2	39	1	2	2	0
5	2	39	2	4	2	39	2	4	3	2	39	2	4	3	0
6	5	39	0	5	5	39	0	0	0	5	39	0	0	0	0
7	3	39	2	5	3	39	2	6	3	3	39	2	6	3	0
8	1	39	5	6	1	39	5	5	5	1	39	5	5	5	0
9	6	38	1	7	6	38	1	6	5	6	38	1	6	6	1
10	12	37	1	13	**11**	37	1	11	10	12	37	1	12	12	2
11	20	38	1	21	20	38	1	20	10	21	39	1	21	10	0
12	2	38	28	30	2	38	28	56	30	2	40	28	56	30	0
13	2	64	4	6	2	64	4	8	8	2	66	4	8	8	0
14	2	68	4	6	2	68	4	8	4	2	70	4	8	4	0
15	38	34	2	40	38	34	2	76	48	38	36	2	76	76	28
16	74	57	17	91	**50**	57	**13**	650	206	74	59	17	1,258	340	134
17	127	40	170	297	127	40	170	21,590	474	151	46	276	41,676	1,348	874
18	2	39	600	602	2	39	600	1,200	97	4	45	1,496	5,984	244	147
19	107	28	4	111	107	28	**3**	321	197	202	36	55	11,110	812	615
20	4	52	278	282	4	52	**276**	1,104	446	8	92	950	7,600	1,483	1,037
21	308	63	131	439	308	63	131	40,348	604	1,139	115	329	374,731	1,931	1,327
22	947	28	0	947	947	28	0	0	0	3,147	38	0	0	0	0
23	256	48	671	927	256	48	671	171,776	188	304	56	2,825	858,800	702	514
24	477	21	151	628	**397**	21	**74**	29,378	1,190	830	24	208	172,640	4,299	3,109
25	31	27	1,678	1,709	31	27	**1,630**	50,530	917	33	28	5,092	168,036	1,318	401
26	29	353	611	640	29	353	**593**	17,197	536	30	431	918	27,540	855	319
PP	-	-	-	945	-	-	-	-	-	-	-	-	-	-	-
$\sum$				7,776				351,501	4,991				1,697,127	13,499	8,508

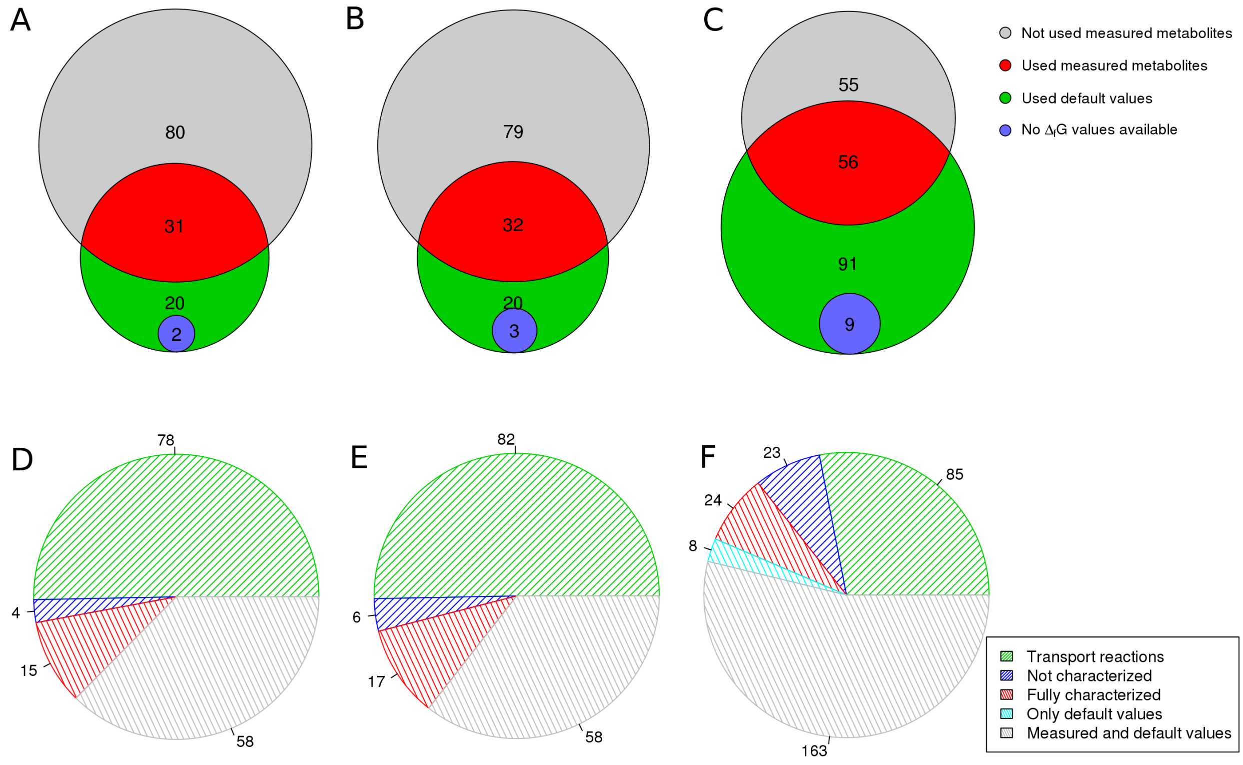

Figure **S1**: Characterization of usable reactions for thermodynamic checks. (A) Out of 111 measured metabolites [3] 31 could be used in the *E. coli* core model M1. For 20 additional metabolites default values were used. Two metabolites, including the biomass, could not be used because of lack of available $\Delta_f G$ value. (B) shows the data for model M2 and (C) for M3. (D) 73 irreversible reactions of the model M1 were used for thermodynamic checks, whereof 15 were fully characterized as for all involved metabolites measured data were available and 58 were partly characterized. 78 transport reactions were not used for the checks as well as 4 uncharacterized reactions, because of missing $\Delta_f G$ information. (E) shows the data for model M2 and (F) for model M3.

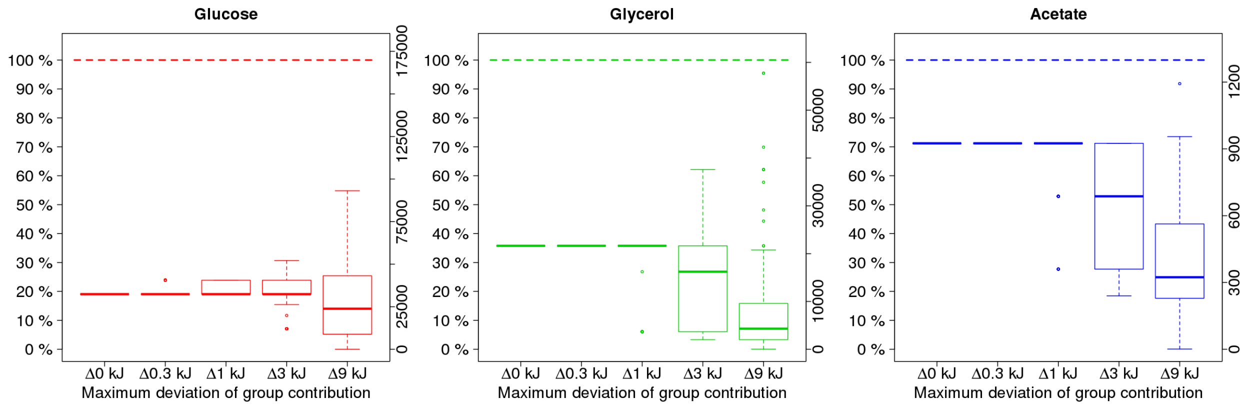

Figure **S2**: The box-plots show the number of calculated EFMs as a function of perturbed $\Delta_f G$ values compared to not perturbed results. Therefore $\Delta_f G$ values for glucose, glycerol and acetate were randomly perturbed by $0, 0.3, 1, 3$ and 9 kJ and EFMs of model M2 were calculated with tEFMA. Each box-plot represents 100 runs.

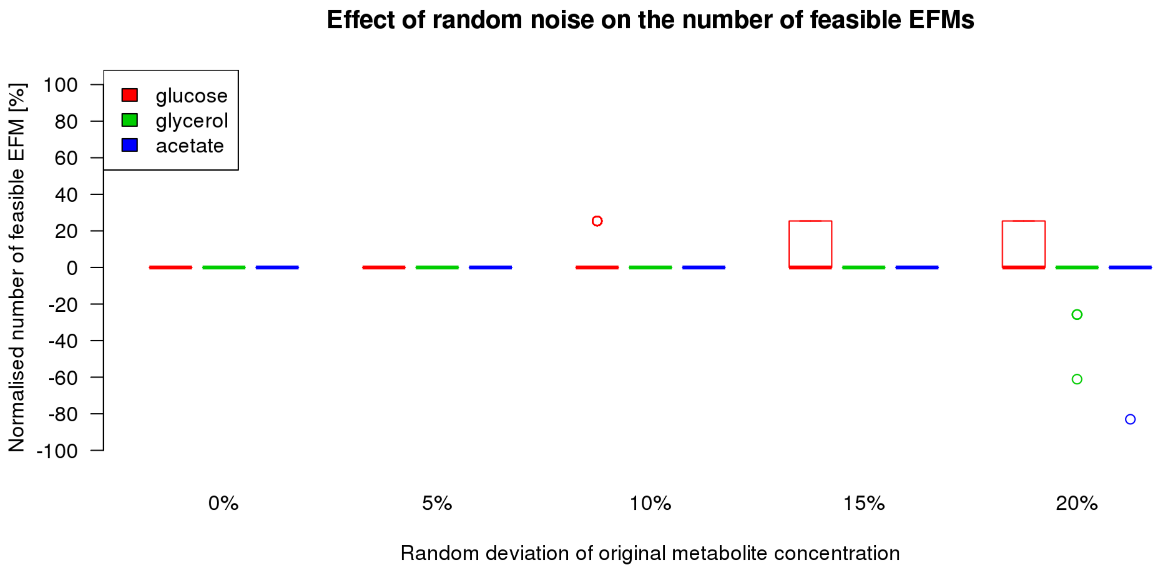

Figure **S3**: Upper and lower metabolite concentration limits for glucose, glycerol and acetate were randomly perturbed by $\pm 5\%$, $\pm 10\%$, $\pm 15\%$ and $\pm 20\%$ and EFMs were calculated. The box-plots show the number of calculated EFMs normalized to not perturbed results averaged over 100 runs each. Note that up to $\pm 15\%$ box-plots are highly degenerated and except for outliers (open circles) only the median value is visible.

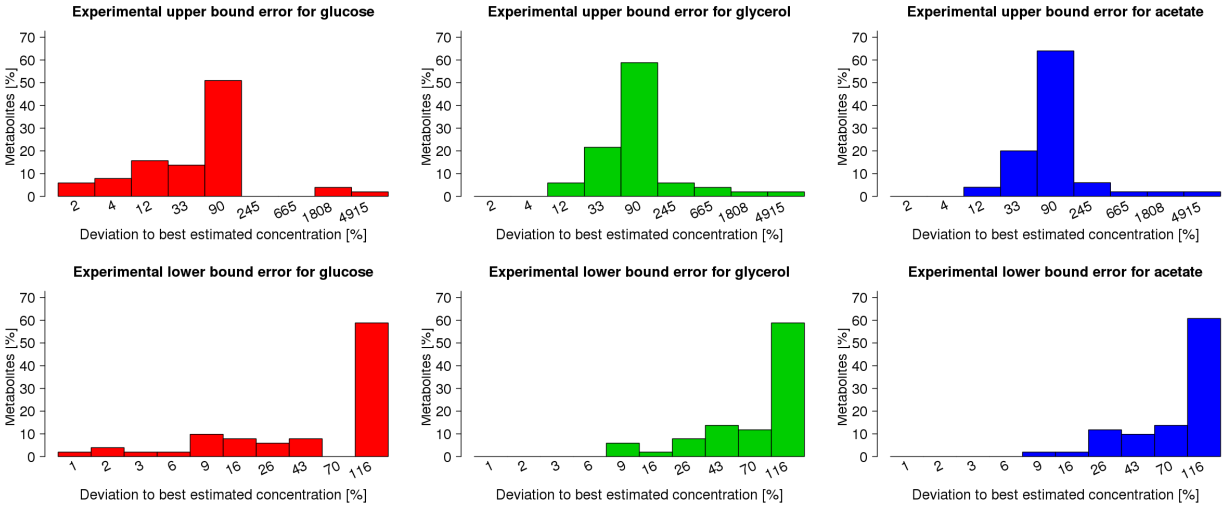

Figure **S4**: Deviation of the upper and lower bound for glucose (left panels), glycerol (middle panels) and acetate (right panels) from the best estimated concentrations as published by [3]. The deviation was calculated by following formula:

$$\text{deviation of metabolite} = \frac{\text{upper or lower concentration}}{\text{best estimated concentration}}$$

The histogram shows the relative number of metabolites as function of the deviation. Note, that the x axis is in logarithmic scale. For all three metabolites, the peak for upper bound concentration is 90% above the best estimated value and below 116% for lower bound concentrations.

Table **S8**: Changes in the upper and lower concentration bounds on glucose (red), glycerol (green), and acetate (blue) upon random perturbations. The full lines illustrate the non-perturbed values illustrated in Figure **S4**.

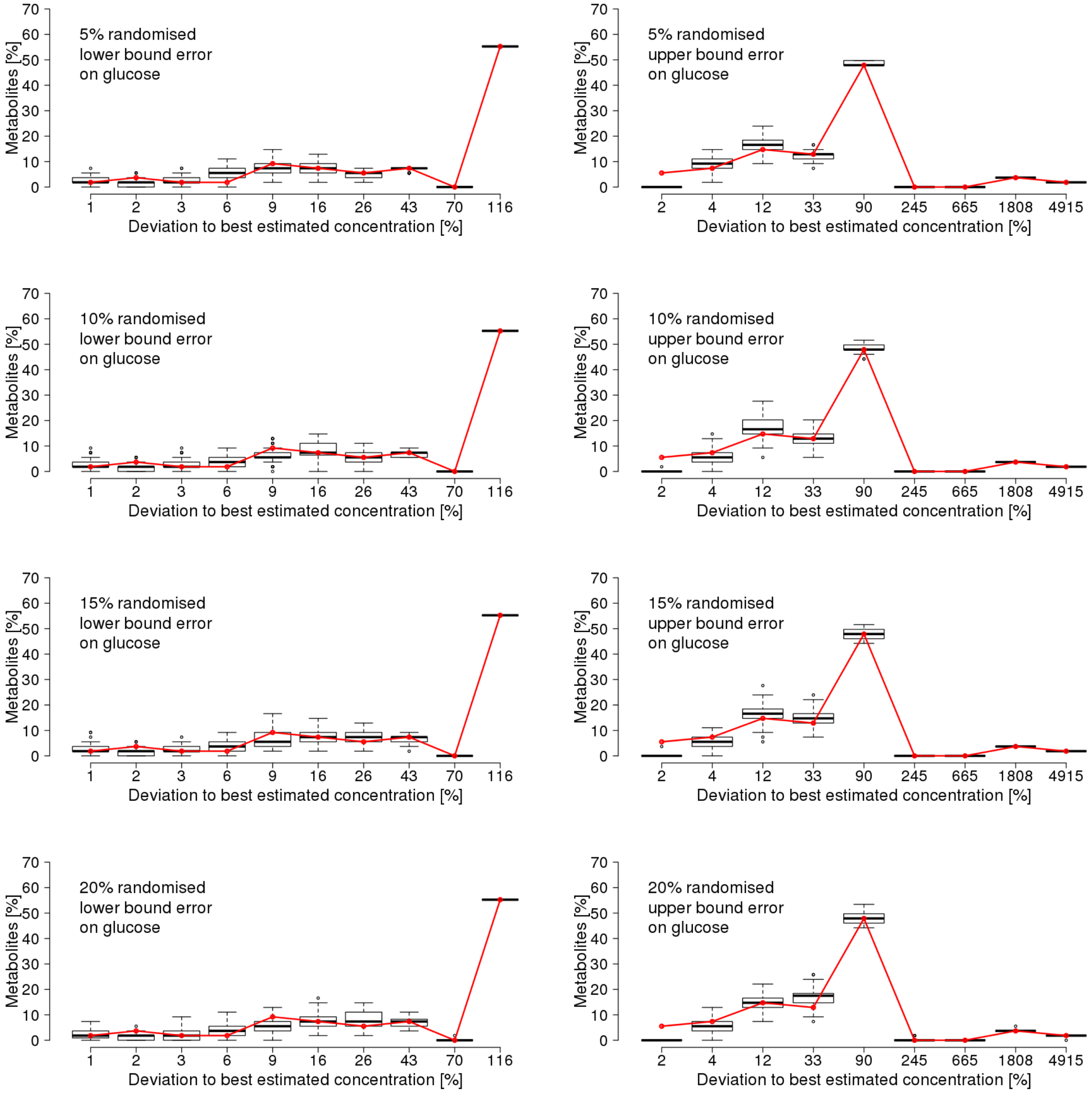

Table **S8** continues on next page.

Table **S8** continues from previous page.

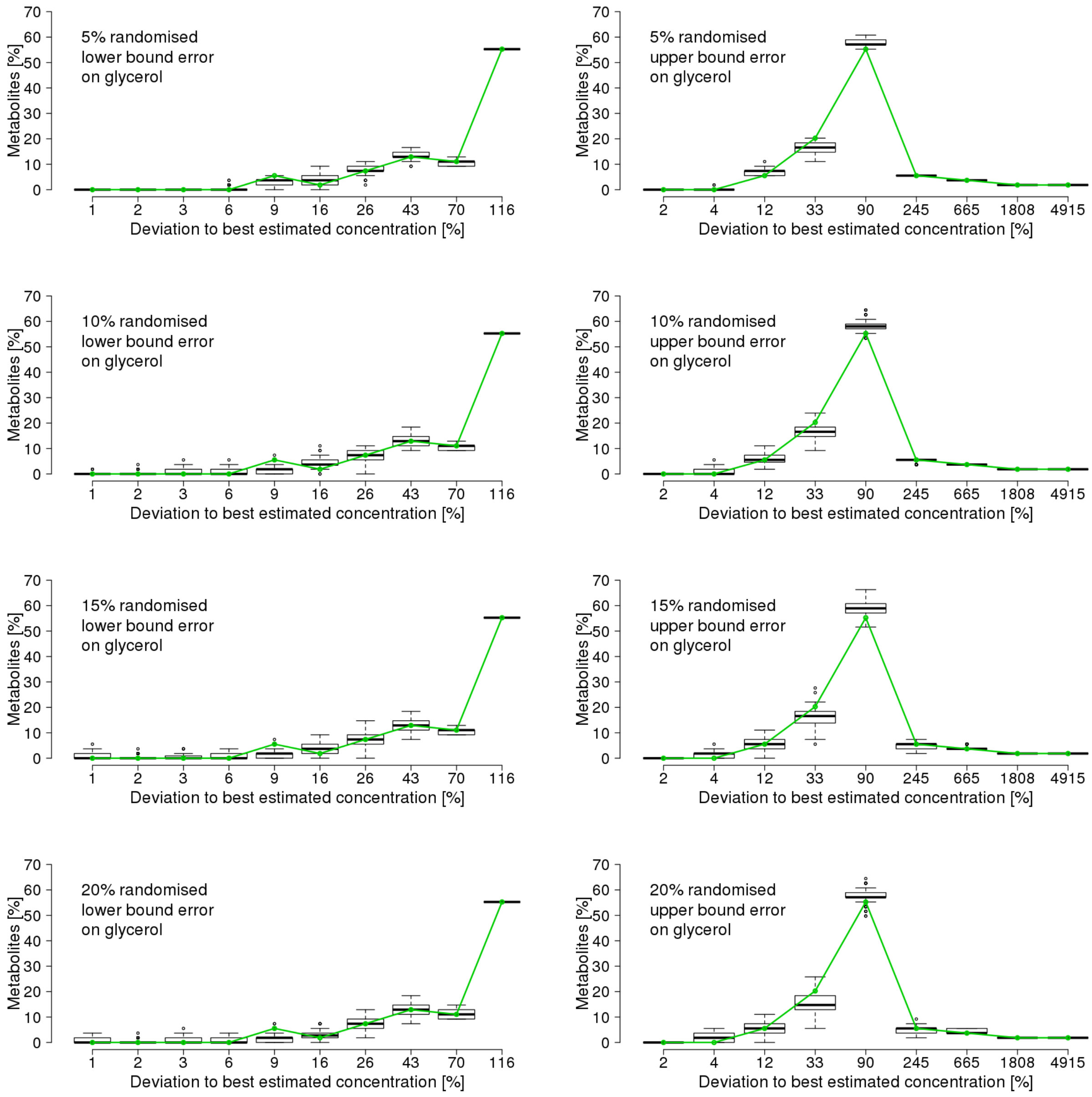

Table **S8** continues on next page.

Table **S8** continues from previous page.

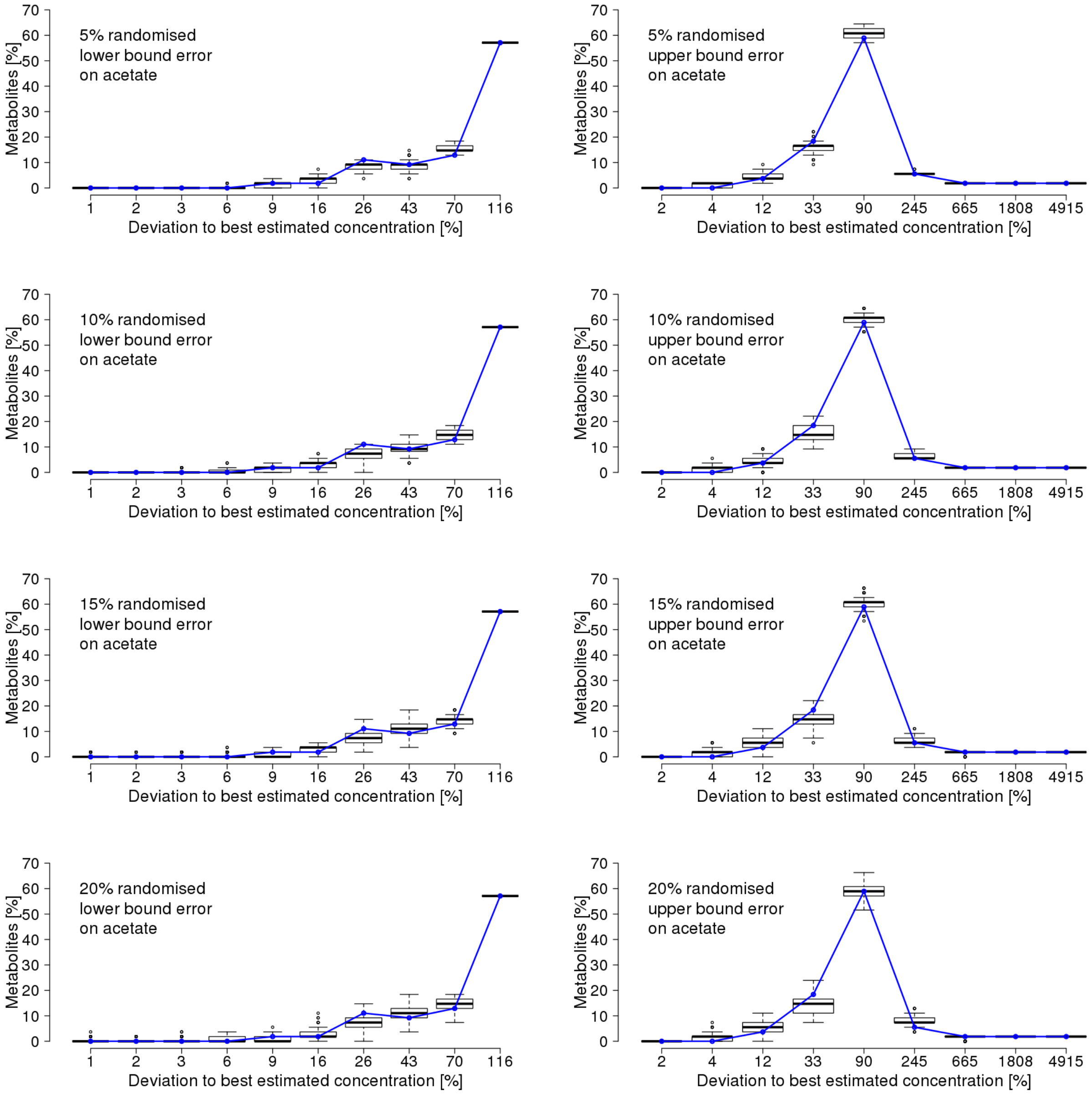

Table **S8** ends.

Table **S9**: Infeasible patterns found in model M2 and M3, when *E. coli* is grown on glucose, acetate or glycerol. The minus in front of the reaction denotes the reverse direction.

M2 on acetate	-ACALD					
	-MDH					
	-TPI	-SUCOAS	PGK	-GAPD		
M2 on glycerol	-ACALD					
	-MDH					
	-ENO	-GLUDy	PGM			
	-FBA	-SUCOAS	-GAPD			
	-FBA	PTAr	PGK	-GAPD		
M2 on glucose	-ACALD					
	-MDH					
	-GLUDy	ACONTb				
	-ENO	-GLUDy	PGM			
	-FBA	-GAPD	PGK			
	-GAPD	-SUCOAS	PGK			
M3 on glucose	-ACALD					
	-MDH					
	-GLUDy	ACONTb				
	-ENO	-GLUDy	PGM			
	-FBA	-GAPD	PGK			
	-GAPD	-SUCOAS	PGK			
	-GLUDy	FUM				
	-MTHFC	FUM				
	-MTHFC	ACONTb				
	-PTAr	-SUCOAS				
	-GAPD	-TPI	PGK			
	-ENO	G5SADs	P5CR	PGM		
	PTAr	ACONTb	G5SADs	G5SD	GLU5K	P5CR
	-SUCOAS	ACONTb	G5SADs	G5SD	GLU5K	P5CR
	-SUCOAS	FUM	G5SADs	G5SD	GLU5K	P5CR

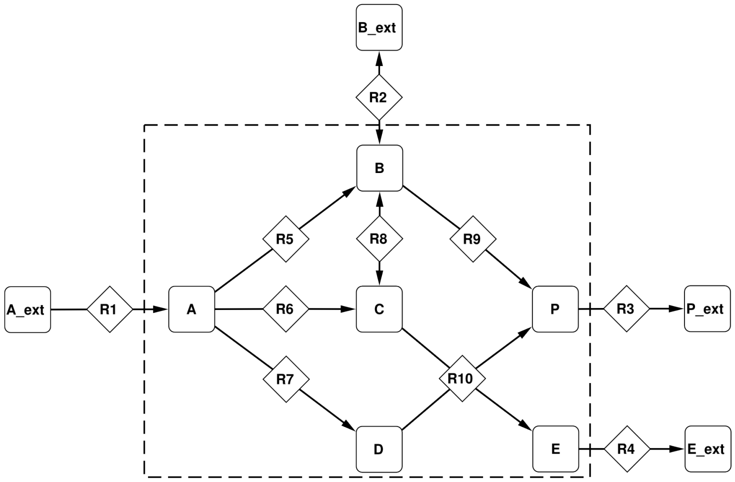

Figure **S5**: Toy network: This network has six internal metabolites (A,B,C,D,E,P) and 10 reactions that are used by *efmtool*. Reactions R_2 and R_8 are reversible.

Proof of safe removal of thermodynamically infeasible EFMs

EFM enumeration by efmtool First, we want to show how EFMs are enumerated by *efmtool*. For this purpose we use the same example as the one in the supplementary material of [4]. Since we want to emphasize on the thermodynamics of EFMs we do not consider the compression methods, adjacency tests and the tree method of the *efmtool* here.

Model

The example is built on the model of [5] (Fig. **S5**) and has the following stoichiometric matrix:

	R_1	R_2	R_3	R_4	R_5	R_6	R_7	R_8	R_9	R_{10}
A	1	0	0	0	-1	-1	-1	0	0	0
B	0	1	0	0	1	0	0	-1	-1	0
C	0	0	0	0	0	1	0	1	0	-1
D	0	0	0	0	0	0	1	0	0	-1
E	0	0	0	-1	0	0	0	0	0	1
P	0	0	-1	0	0	0	0	0	1	1

The rows of the stoichiometric matrix represent the internal metabolites (A,B,C,D,E,P) and the columns the reactions ($R_1, R_2, R_3, R_4, R_5, R_6, R_7, R_8, R_9, R_{10}$).

Initialization phase

The algorithm starts with the initialization phase and splits the reversible reactions (R_2 and R_8) into two irreversible reactions each.

	R_1	R_2	R_{2r}	R_3	R_4	R_5	R_6	R_7	R_8	R_{8r}	R_9	R_{10}
A	1	0	0	0	0	−1	−1	−1	0	0	0	0
B	0	1	−1	0	0	1	0	0	−1	1	−1	0
C	0	0	0	0	0	0	1	0	1	−1	0	−1
D	0	0	0	0	0	0	0	1	0	0	0	−1
E	0	0	0	0	−1	0	0	0	0	0	0	1
P	0	0	0	−1	0	0	0	0	0	0	1	1

In the next step the kernel matrix is computed and then the rows are permuted

$$
\begin{array}{l|rrrrrr}
R_1 & 0 & 1 & -1 & 1 & 0 & 2 \\
R_2 & 1 & -1 & 1 & -1 & 1 & 0 \\
R_{2r} & 1 & 0 & 0 & 0 & 0 & 0 \\
R_3 & 0 & 0 & 0 & 0 & 1 & 1 \\
R_4 & 0 & 0 & 0 & 0 & 0 & 1 \\
R_5 & 0 & 1 & 0 & 0 & 0 & 0 \\
R_6 & 0 & 0 & -1 & 1 & 0 & 1 \\
R_7 & 0 & 0 & 0 & 0 & 0 & 1 \\
R_8 & 0 & 0 & 1 & 0 & 0 & 0 \\
R_{8r} & 0 & 0 & 0 & 1 & 0 & 0 \\
R_9 & 0 & 0 & 0 & 0 & 1 & 0 \\
R_{10} & 0 & 0 & 0 & 0 & 0 & 1
\end{array}
\quad \Rightarrow \quad
\begin{array}{l|rrrrrr}
R_{2r} & 1 & 0 & 0 & 0 & 0 & 0 \\
R_5 & 0 & 1 & 0 & 0 & 0 & 0 \\
R_8 & 0 & 0 & 1 & 0 & 0 & 0 \\
R_{8r} & 0 & 0 & 0 & 1 & 0 & 0 \\
R_9 & 0 & 0 & 0 & 0 & 1 & 0 \\
R_4 & 0 & 0 & 0 & 0 & 0 & 1 \\
R_{10} & 0 & 0 & 0 & 0 & 0 & 1 \\
R_7 & 0 & 0 & 0 & 0 & 0 & 1 \\
R_3 & 0 & 0 & 0 & 0 & 1 & 1 \\
R_6 & 0 & 0 & -1 & 1 & 0 & 1 \\
R_1 & 0 & 1 & -1 & 1 & 0 & 2 \\
R_2 & 1 & -1 & 1 & -1 & 1 & 0
\end{array}
$$

Iterations and thermodynamic feasibility checks

In the iteration phase all EFMs are enumerated. The EFMs are represented by the columns of the generated matrix. Here, in the first step the first nine lines are easily converted to binary values as they contain no negative values. We use $\bullet$ for reactions carrying a flux and $\star$ for non active

reactions in the binary notation (note that [4] use the inverse logic in their publication).

R_{2r}	•	⋆	⋆	⋆	⋆	⋆
R_5	⋆	•	⋆	⋆	⋆	⋆
R_8	⋆	⋆	•	⋆	⋆	⋆
R_{8r}	⋆	⋆	⋆	•	⋆	⋆
R_9	⋆	⋆	⋆	⋆	•	⋆
R_4	⋆	⋆	⋆	⋆	⋆	•
R_{10}	⋆	⋆	⋆	⋆	⋆	•
R_7	⋆	⋆	⋆	⋆	⋆	•
R_3	⋆	⋆	⋆	⋆	•	•
R_6	0	0	−1	1	0	1
R_1	0	1	−1	1	0	2
R_2	1	−1	1	−1	1	0

At the start of each iteration the binary part of modes with positive or negative values on the next numeric position (R_6) is checked for thermodynamic feasibility. For this purpose a linear problem is created containing constraints for all reactions in a column carrying a flux (denoted with •). In this example we assume that it is thermodynamically not feasible that R_8 and R_{10} are active together in the same EFM. In other words, it is not allowed that both reactions are labeled with • in the same column. As none of the modes are infeasible so far we proceed with the next iteration.

As the next row has one negative value in column three the corresponding mode (column) is removed and combined with all modes containing a positive value (column 4 and 6). The combination is performed by a logic OR which is described by the following truth table:

Table **S10**: Truth table for the combination in *efmtool*

column A	column B	new column
$\bullet$	$\bullet$	$\bullet$
$\bullet$	$\star$	$\bullet$
$\star$	$\bullet$	$\bullet$
$\star$	$\star$	$\star$

Without thermodynamic check

R_{2r}	$\bullet$	$\star$	$\star$	$\star$	$\star$	$\star$	$\star$
R_5	$\star$	$\bullet$	$\star$	$\star$	$\star$	$\star$	$\star$
R_8	$\star$	$\star$	$\star$	$\star$	$\star$	$\bullet$	$\bullet$
R_{8r}	$\star$	$\star$	$\bullet$	$\star$	$\star$	$\bullet$	$\star$
R_9	$\star$	$\star$	$\star$	$\bullet$	$\star$	$\star$	$\star$
R_4	$\star$	$\star$	$\star$	$\star$	$\bullet$	$\star$	$\bullet$
R_{10}	$\star$	$\star$	$\star$	$\star$	$\bullet$	$\star$	$\bullet$
R_7	$\star$	$\star$	$\star$	$\star$	$\bullet$	$\star$	$\bullet$
R_3	$\star$	$\star$	$\star$	$\bullet$	$\bullet$	$\star$	$\bullet$
R_6	$\star$	$\star$	$\bullet$	$\star$	$\bullet$	$\star$	$\star$
R_1	0	1	1	0	2	0	1
R_2	1	−1	−1	1	0	0	1

With thermodynamic check

R_{2r}	$\bullet$	$\star$	$\star$	$\star$	$\star$	$\star$
R_5	$\star$	$\bullet$	$\star$	$\star$	$\star$	$\star$
R_8	$\star$	$\star$	$\star$	$\star$	$\star$	$\bullet$
R_{8r}	$\star$	$\star$	$\bullet$	$\star$	$\star$	$\bullet$
R_9	$\star$	$\star$	$\star$	$\bullet$	$\star$	$\star$
R_4	$\star$	$\star$	$\star$	$\star$	$\bullet$	$\star$
R_{10}	$\star$	$\star$	$\star$	$\star$	$\bullet$	$\star$
R_7	$\star$	$\star$	$\star$	$\star$	$\bullet$	$\star$
R_3	$\star$	$\star$	$\star$	$\bullet$	$\bullet$	$\star$
R_6	$\star$	$\star$	$\bullet$	$\star$	$\bullet$	$\star$
R_1	0	1	1	0	2	0
R_2	1	−1	−1	1	0	0

In the left table all modes after this iteration step are shown. In the last column, R_8 and R_{10} are both marked with $\bullet$. Therefore this mode is thermodynamically infeasible. As this mode has a positive value in row R_1, it is checked at the start of the next iteration and removed immediately (right table).

The next step is again a simple conversion from numeric to binary values as there are no negative values in row R_1.

Without thermodynamic check

R_{2r}	•	⋆	⋆	⋆	⋆	⋆	⋆
R_5	⋆	•	⋆	⋆	⋆	⋆	⋆
R_8	⋆	⋆	⋆	⋆	⋆	•	•
R_{8r}	⋆	⋆	•	⋆	⋆	•	⋆
R_9	⋆	⋆	⋆	•	⋆	⋆	⋆
R_4	⋆	⋆	⋆	⋆	•	⋆	•
R_{10}	⋆	⋆	⋆	⋆	•	⋆	•
R_7	⋆	⋆	⋆	⋆	•	⋆	•
R_3	⋆	⋆	⋆	•	•	⋆	•
R_6	⋆	⋆	•	⋆	•	⋆	⋆
R_1	⋆	•	•	⋆	•	⋆	•
R_2	1	−1	−1	1	0	0	1

With thermodynamic check

R_{2r}	•	⋆	⋆	⋆	⋆	⋆
R_5	⋆	•	⋆	⋆	⋆	⋆
R_8	⋆	⋆	⋆	⋆	⋆	•
R_{8r}	⋆	⋆	•	⋆	⋆	•
R_9	⋆	⋆	⋆	•	⋆	⋆
R_4	⋆	⋆	⋆	⋆	•	⋆
R_{10}	⋆	⋆	⋆	⋆	•	⋆
R_7	⋆	⋆	⋆	⋆	•	⋆
R_3	⋆	⋆	⋆	•	•	⋆
R_6	⋆	⋆	•	⋆	•	⋆
R_1	⋆	•	•	⋆	•	⋆
R_2	1	−1	−1	1	0	0

In the next step two columns with negative values (2 and 3) have to be combined with positive columns (1,4,7 in left table and 1,4 in right table).

Without thermodynamic check

R_{2r}	$\bullet$	$\star$	$\star$	$\star$	$\star$	$\bullet$	$\bullet$	$\star$	$\star$	$\star$
R_5	$\star$	$\star$	$\star$	$\star$	$\star$	$\bullet$	$\star$	$\bullet$	$\bullet$	$\star$
R_8	$\star$	$\star$	$\star$	$\bullet$	$\bullet$	$\star$	$\star$	$\bullet$	$\star$	$\star$
R_{8r}	$\star$	$\star$	$\star$	$\bullet$	$\star$	$\star$	$\bullet$	$\star$	$\star$	$\bullet$
R_9	$\star$	$\bullet$	$\star$	$\star$	$\star$	$\star$	$\star$	$\star$	$\bullet$	$\bullet$
R_4	$\star$	$\star$	$\bullet$	$\star$	$\bullet$	$\star$	$\star$	$\bullet$	$\star$	$\star$
R_{10}	$\star$	$\star$	$\bullet$	$\star$	$\bullet$	$\star$	$\star$	$\bullet$	$\star$	$\star$
R_7	$\star$	$\star$	$\bullet$	$\star$	$\bullet$	$\star$	$\star$	$\bullet$	$\star$	$\star$
R_3	$\star$	$\bullet$	$\bullet$	$\star$	$\bullet$	$\star$	$\star$	$\bullet$	$\bullet$	$\bullet$
R_6	$\star$	$\star$	$\bullet$	$\star$	$\star$	$\star$	$\bullet$	$\star$	$\star$	$\bullet$
R_1	$\star$	$\star$	$\bullet$	$\star$	$\bullet$	$\bullet$	$\bullet$	$\bullet$	$\bullet$	$\bullet$
R_2	$\bullet$	$\bullet$	$\star$	$\star$	$\bullet$	$\star$	$\star$	$\star$	$\star$	$\star$

With thermodynamic check

R_{2r}	$\bullet$	$\star$	$\star$	$\star$	$\bullet$	$\bullet$	$\star$	$\star$
R_5	$\star$	$\star$	$\star$	$\star$	$\bullet$	$\star$	$\bullet$	$\star$
R_8	$\star$	$\star$	$\star$	$\bullet$	$\star$	$\star$	$\star$	$\star$
R_{8r}	$\star$	$\star$	$\star$	$\bullet$	$\star$	$\bullet$	$\star$	$\bullet$
R_9	$\star$	$\bullet$	$\star$	$\star$	$\star$	$\star$	$\bullet$	$\bullet$
R_4	$\star$	$\star$	$\bullet$	$\star$	$\star$	$\star$	$\star$	$\star$
R_{10}	$\star$	$\star$	$\bullet$	$\star$	$\star$	$\star$	$\star$	$\star$
R_7	$\star$	$\star$	$\bullet$	$\star$	$\star$	$\star$	$\star$	$\star$
R_3	$\star$	$\bullet$	$\bullet$	$\star$	$\star$	$\star$	$\bullet$	$\bullet$
R_6	$\star$	$\star$	$\bullet$	$\star$	$\star$	$\bullet$	$\star$	$\bullet$
R_1	$\star$	$\star$	$\bullet$	$\star$	$\bullet$	$\bullet$	$\bullet$	$\bullet$
R_2	$\bullet$	$\bullet$	$\star$	$\star$	$\star$	$\star$	$\star$	$\star$

In the left table two new thermodynamically infeasible EFMs which do not appear in the right table are created.

Postprocessing phase

In the postprocessing step of *efmtool* the rows of the matrix are reordered to restore the original order of the reactions:

Without thermodynamic check

R_1	$\star$	$\star$	$\bullet$	$\star$	$\bullet$	$\bullet$	$\bullet$	$\bullet$	$\bullet$	$\bullet$
R_2	$\bullet$	$\bullet$	$\star$	$\star$	$\bullet$	$\star$	$\star$	$\star$	$\star$	$\star$
R_{2r}	$\bullet$	$\star$	$\star$	$\star$	$\star$	$\bullet$	$\bullet$	$\star$	$\star$	$\star$
R_3	$\star$	$\bullet$	$\bullet$	$\star$	$\bullet$	$\star$	$\star$	$\bullet$	$\bullet$	$\bullet$
R_4	$\star$	$\star$	$\bullet$	$\star$	$\bullet$	$\star$	$\star$	$\bullet$	$\star$	$\star$
R_5	$\star$	$\star$	$\star$	$\star$	$\star$	$\bullet$	$\star$	$\bullet$	$\bullet$	$\star$
R_6	$\star$	$\star$	$\bullet$	$\star$	$\star$	$\star$	$\bullet$	$\star$	$\star$	$\bullet$
R_7	$\star$	$\star$	$\bullet$	$\star$	$\bullet$	$\star$	$\star$	$\bullet$	$\star$	$\star$
R_8	$\star$	$\star$	$\star$	$\bullet$	$\bullet$	$\star$	$\star$	$\bullet$	$\star$	$\star$
R_{8r}	$\star$	$\star$	$\star$	$\bullet$	$\star$	$\star$	$\bullet$	$\star$	$\star$	$\bullet$
R_9	$\star$	$\bullet$	$\star$	$\star$	$\star$	$\star$	$\star$	$\star$	$\bullet$	$\bullet$
R_{10}	$\star$	$\star$	$\bullet$	$\star$	$\bullet$	$\star$	$\star$	$\bullet$	$\star$	$\star$

With thermodynamic check

R_1	$\star$	$\star$	$\bullet$	$\star$	$\bullet$	$\bullet$	$\bullet$	$\bullet$
R_2	$\bullet$	$\bullet$	$\star$	$\star$	$\star$	$\star$	$\star$	$\star$
R_{2r}	$\bullet$	$\star$	$\star$	$\star$	$\bullet$	$\bullet$	$\star$	$\star$
R_3	$\star$	$\bullet$	$\bullet$	$\star$	$\star$	$\star$	$\bullet$	$\bullet$
R_4	$\star$	$\star$	$\bullet$	$\star$	$\star$	$\star$	$\star$	$\star$
R_5	$\star$	$\star$	$\star$	$\star$	$\bullet$	$\star$	$\bullet$	$\star$
R_6	$\star$	$\star$	$\bullet$	$\star$	$\star$	$\bullet$	$\star$	$\bullet$
R_7	$\star$	$\star$	$\bullet$	$\star$	$\star$	$\star$	$\star$	$\star$
R_8	$\star$	$\star$	$\star$	$\bullet$	$\star$	$\star$	$\star$	$\star$
R_{8r}	$\star$	$\star$	$\star$	$\bullet$	$\star$	$\bullet$	$\star$	$\bullet$
R_9	$\star$	$\bullet$	$\star$	$\star$	$\star$	$\star$	$\bullet$	$\bullet$
R_{10}	$\star$	$\star$	$\bullet$	$\star$	$\star$	$\star$	$\star$	$\star$

In the next step the binary values are converted back to numeric values:

Without thermodynamic check

R_1	0	0	2	0	1	1	1	2	1	1
R_2	1	1	0	0	1	0	0	0	0	0
R_{2r}	1	0	0	0	0	1	1	0	0	0
R_3	0	1	1	0	1	0	0	1	1	1
R_4	0	0	1	0	1	0	0	1	0	0
R_5	0	0	0	0	0	1	0	1	1	0
R_6	0	0	1	0	0	0	1	0	0	1
R_7	0	0	1	0	1	0	0	1	0	0
R_8	0	0	0	1	1	0	0	1	0	0
R_{8r}	0	0	0	1	0	0	1	0	0	1
R_9	0	1	0	0	0	0	0	0	1	1
R_{10}	0	0	1	0	1	0	0	1	0	0

With thermodynamic check

R_1	0	0	2	0	1	1	1	1
R_2	1	1	0	0	0	0	0	0
R_{2r}	1	0	0	0	1	1	0	0
R_3	0	1	1	0	0	0	1	1
R_4	0	0	1	0	0	0	0	0
R_5	0	0	0	0	1	0	1	0
R_6	0	0	1	0	0	1	0	1
R_7	0	0	1	0	0	0	0	0
R_8	0	0	0	1	0	0	0	0
R_{8r}	0	0	0	1	0	1	0	1
R_9	0	1	0	0	0	0	1	1
R_{10}	0	0	1	0	0	0	0	0

Rows representing reversible reactions are combined and modes with futile cycles (1. and 4. column) are removed.

Without thermodynamic check

	EM_1	EM_2	EM_3	EM_4	EM_5	EM_6	EM_7	EM_8
R_1	0	2	1	1	1	2	1	1
R_2	1	0	1	−1	−1	0	0	0
R_3	1	1	1	0	0	1	1	1
R_4	0	1	1	0	0	1	0	0
R_5	0	0	0	1	0	1	1	0
R_6	0	1	0	0	1	0	0	1
R_7	0	1	1	0	0	1	0	0
R_8	0	0	1	0	−1	1	0	−1
R_9	1	0	0	0	0	0	1	1
R_{10}	0	1	1	0	0	1	0	0

With thermodynamic check

	EM_1	EM_2	EM_4	EM_5	EM_7	EM_8
R_1	0	2	1	1	1	1
R_2	1	0	−1	−1	0	0
R_3	1	1	0	0	1	1
R_4	0	1	0	0	0	0
R_5	0	0	1	0	1	0
R_6	0	1	0	1	0	1
R_7	0	1	0	0	0	0
R_8	0	0	0	−1	0	−1
R_9	1	0	0	0	1	1
R_{10}	0	1	0	0	0	0

The toy network results in 8 EFMs(left table), whereas in this example EFM_3 and EFM_6 are thermodynamically infeasible (right table).

* Feasible EFMs are never removed As shown before, new EFMs are generated by a combination of previously calculated EFMs. Here we show that thermodynamically infeasible modes can never contribute to new thermodynamically feasible modes and can safely be removed during the iteration phase.

- In the main part of this paper we have shown that the constraints of the linear program for checking the feasibility of an EFM are defined by all reactions carrying a flux in this mode.

- Here, a reaction carrying a flux is marked by •
- It is shown in the truth table (Table **S10**) that an active reaction will always stay active by the combination of adjacent candidates, as it is done by *efmtool*.
- Hence, a combined mode always contains all active reactions of the two single contributing modes.
- It is not possible to turn an infeasible flux into a feasible flux by adding additional reactions.
- Thus, a combination of an infeasible EFM (e.g. defined by NET analysis) with any other EFM will always result in an infeasible EFM and can therefore be removed without loss of feasible EFMs.

Calculation of the transformed standard Gibbs free energy of formation

$$\Delta_\mathrm{f} G'^0 = -\mathrm{R}T \ln \left\{ \sum_i \exp\left[-\Delta_\mathrm{f} G_i^0(pH, I)/\mathrm{R}T\right] \right\} \tag{1a}$$

$$\Delta_\mathrm{f} G_i^0(pH, I) = \Delta_\mathrm{f} G_i^0 - \Delta_\mathrm{f} G_i^0(pH) - \Delta_\mathrm{f} G_i^0(I) \tag{1b}$$

$$\Delta_\mathrm{f} G_i^0(pH) = H_i^+ \mathrm{R}T \ln\left(10^{-pH}\right) \tag{1c}$$

$$\Delta_\mathrm{f} G_i^0(I) = \sqrt{I}\mathrm{A}\frac{z_i^2 - H_i^+}{1 + \sqrt{I}\mathrm{B}} \tag{1d}$$

$$\mathrm{A} = 2.91482\,\mathrm{kJ\,mol^{-1}\,M^{-0.5}}, \quad \mathrm{B} = 1.6\,\mathrm{M^{-0.5}} \tag{1e}$$

where the summation over i has to be carried out over all net charges, z_i, of a metabolite. $\Delta_\mathrm{f} G_i^0(pH, I)$ denotes the standard transformed Gibbs free energy of formation for the metabolite corrected for

ionic strength, I [$\Delta_\mathrm{f} G_i^0(I)$], and pH [$\Delta_\mathrm{f} G_i^0(pH)$] at charge state z_i. The standard Gibbs free energy of formation, $\Delta_\mathrm{f} G_i^0$, was estimated using the online version of eQuilibrator [6]. Finally, H^+ denotes the number of protons while A and B are constants [7]. We set

$$I = 0.15\,\mathrm{M}, \quad pH = 7, \quad \text{and} \quad T = 310.15\,\mathrm{K}\,(37\,^\circ\mathrm{C}). \tag{2}$$

Values for the metabolite's $\Delta_\mathrm{f} G'^0$ are listed in the supplementary material, file 2.

1. Smoot, M. E., Ono, K., Ruscheinski, J., Wang, P.-L. & Ideker, T. Cytoscape 2.8: new features for data integration and network visualization. *Bioinformatics* **27**, 431–432; DOI:10.1093/bioinformatics/btq675 (2011).

2. Orth, J. D., Fleming, R. M. T. & Palsson, B. Ø. Reconstruction and Use of Microbial Metabolic Networks: the Core Escherichia coli Metabolic Model as an Educational Guide. *EcoSal* (2010).

3. Bennett, B. D. *et al.* Absolute metabolite concentrations and implied enzyme active site occupancy in Escherichia coli. *Nat. Chem. Biol.* **5**, 593–599; DOI:10.1038/nchembio.186 (2009).

4. Terzer, M. & Stelling, J. Large-scale computation of elementary flux modes with bit pattern trees. *Bioinformatics* **24**, 2229 –2235; DOI:10.1093/bioinformatics/btn401 (2008).

5. Klamt, S. & Stelling, J. *Stoichiometric and constraint-based modeling*, 73–96; DOI:10.7551/mitpress/9780262195485.003.0005 (MIT Press (Cambridge / MA), 2006).

6. Flamholz, A., Noor, E., Bar-Even, A. & Milo, R. eQuilibrator–the biochemical thermodynamics calculator. *Nucleic. Acids. Res.* **40**, D770–D775; DOI:10.1093/nar/gkr874 (2012).

7. Alberty, R. A. *Thermodynamics of Biochemical Reactions* (John Wiley & Sons, Inc., New Jersey, 2003).
